# Effect of sodium-glucose cotransporter-2 inhibitors on haemoglobin and haematocrit levels in heart failure: a systematic review and meta-analysis

**DOI:** 10.1093/eschf/xvag027

**Published:** 2026-01-19

**Authors:** Shiva Armani Moghadam, Amirreza Shahmohammadi, Keyvan Salehi, Roozbeh Narimani-Javid, Parnian Soltani, Sahar Zafarmandi, Mohammad Hossein Shafieyoun, Mehrdad Mahalleh, Raheel Ahmed, Tarek Bekfani, Tor Biering-Sørensen, Kaveh Hosseini

**Affiliations:** Heart Failure Research Center, Cardiovascular Research Institute, Isfahan University of Medical Sciences, Isfahan, Iran; Research Center for Advanced Technologies in Cardiovascular Medicine, Cardiovascular Diseases Research Institute, Tehran University of Medical Sciences, Tehran, Iran; School of Medicine, Shahid Beheshti University of Medical Science, Tehran, Iran; Tehran Heart Center, Cardiovascular Diseases Research Institute, Tehran University of Medical Sciences, Tehran, Iran; Research Center for Advanced Technologies in Cardiovascular Medicine, Cardiovascular Diseases Research Institute, Tehran University of Medical Sciences, Tehran, Iran; Tehran Heart Center, Cardiovascular Diseases Research Institute, Tehran University of Medical Sciences, Tehran, Iran; Tehran Heart Center, Cardiovascular Diseases Research Institute, Tehran University of Medical Sciences, Tehran, Iran; Isfahan Cardiovascular Research Center, Cardiovascular Research Institute, Isfahan University of Medical Sciences, Isfahan, Iran; Research Center for Advanced Technologies in Cardiovascular Medicine, Cardiovascular Diseases Research Institute, Tehran University of Medical Sciences, Tehran, Iran; Academic Clinical Lecturer, Newcastle University, Newcastle, UK; Department of Cardiology, Freeman Hospital, Newcastle upon Tyne, UK; Department of Cardiology, Royal Brompton Hospital, London, UK; Department of Cardiology, University Hospital Magdeburg, Magdeburg, Germany; Department of Cardiology, Copenhagen University Hospital—Herlev and Gentofte, Copenhagen, Denmark; Center for Translational Cardiology and Pragmatic Randomized Trials, Department of Biomedical Sciences, Faculty of Health and Medical Sciences, University of Copenhagen, Copenhagen, Denmark; Tehran Heart Center, Cardiovascular Diseases Research Institute, Tehran University of Medical Sciences, Tehran, Iran; Center for Translational Cardiology and Pragmatic Randomized Trials, Department of Biomedical Sciences, Faculty of Health and Medical Sciences, University of Copenhagen, Copenhagen, Denmark; Department of Cardiology, Copenhagen University Hospital—Herlev and Gentofte, Copenhagen, Denmark

**Keywords:** Heart failure, SGLT2 inhibitors, Haemoglobin, Haematocrit, Meta-analysis

## Abstract

The aim of this study is to evaluate the effects of sodium-glucose cotransporter-2 (SGLT2) inhibitors on haemoglobin (Hb) and haematocrit (Hct) levels in patients with heart failure (HF). We systematically searched PubMed, Web of Science, Cochrane Library, and Embase for randomized controlled trials (RCTs) until April 2025. Changes in Hb and Hct were evaluated in HF patients treated with SGLT2 inhibitors compared to control subjects. A random-effects model was applied to calculate mean differences (MDs) with corresponding 95% confidence intervals (CIs). Subgroup analyses were performed across different SGLT2 inhibitors, follow-up durations, and types of control groups. Between-study heterogeneity was quantified using the I^2^ statistic, and meta-regression analyses were performed to explore the influence of baseline clinical characteristics on haematologic responses. Publication bias was evaluated using funnel plots and Egger’s test. Seventeen randomized controlled trials (RCTs) with 16 784 participants (mean age 68.65 years, 65.56% male) were included. Sodium-glucose cotransporter-2 inhibitors significantly increased Hb (MD = 0.68 g/dl, 95% CI: 0.53; 0.83, *I*^2^ = 39.7%, *P*-value <.0001) and Hct (MD = 2.15%, 95% CI: 1.73; 2.57, *I*^2^ = 66.6%, *P*-value < .0001) compared with controls. Subgroup analyses showed consistent benefits across individual SGLT2 inhibitors, duration of follow-up (≥6 months vs <6 months), and comparator type (placebo vs active control). There was no evidence of publication bias. Sodium-glucose cotransporter-2 inhibitors significantly increase levels of Hb and Hct in patients with HF. Further research is warranted to assess clinical significance in relation to Hb and Hct changes in patients with pre-existing anaemia or renal dysfunction.

## Graphical Abstract

**Graphical Abstract:**
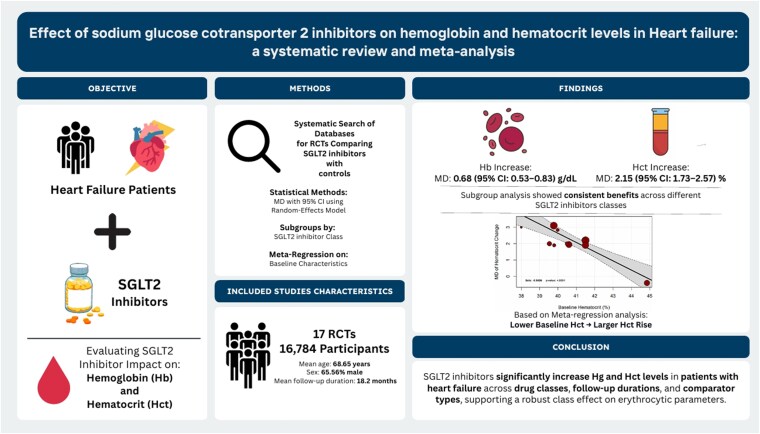
Metaanlysis overview.

## Introduction

Anaemia is a frequent comorbidity in heart failure (HF), and studies show that this condition impacts HF management and outcomes.^1–3^ In patients with HF, it is linked to elevated hospitalization rates, reduced functional capacity, and increased mortality.^4,5^ Therefore, anaemia in HF is considered an important clinical concern; however, large trials evaluating the correction of HF-related anaemia have demonstrated mixed effects on hard composite clinical outcomes.^6–8^ While anaemia is increasingly considered a marker of HF severity rather than a proven therapeutic target, its presence remains relevant to patients’ symptoms and risk stratification.^7,9^

Sodium-glucose cotransporter-2 (SGLT2) inhibitors are primarily indicated for patients diagnosed with diabetes mellitus (DM) and have been shown to confer substantial cardiovascular and renal protection in addition to their glucose-lowering effects.^10,11^ Clinically, these drugs lower the risk of worsening HF and cardiovascular mortality and reduce HF hospitalization^12^ (CKD) progression, and mitigating renal oxidative stress.^13,14^ Notably, analysis from the EMPA-REG OUTCOME trial in patients with Type 2 diabetes indicated that Empagliflozin's effect on reducing the risk of cardiovascular death compared to placebo was 51.8% due to changes in haematocrit (Hct) and 48.9% due to changes in Hb, based on differences from baseline.^15^ This highlights the importance of Hb and Hct correction in reducing cardiovascular risks among patients treated with SGLT2 inhibitors.

Despite these findings, the impact of SGLT2 inhibitors on Hb and Hct among individuals with HF remains uncertain. Most systematic reviews have centred on diabetic populations,^16,17^ so their conclusions may not fully apply to HF. This highlights the need for a dedicated synthesis of evidence in this group.

Moreover, since SGLT2 inhibitors are now recommended across all stages of HF, including HF with reduced ejection fraction (HFrEF), preserved ejection fraction (HFpEF), and mildly reduced ejection fraction (HFmrEF),^18^ it is important to understand whether their haematologic effects are consistent across these subgroups. Differences in pathophysiology and baseline anaemia prevalence between these HF phenotypes may influence the magnitude and clinical relevance of Hb and Hct changes.^19^ This systematic review and meta-analysis aims to evaluate the haematologic effects of SGLT2 inhibitors in HF patients by synthesizing the available evidence on their impact on Hb and Hct levels.

## Method

### Protocol and registration

This systematic review and meta-analysis adhered to the preferred reporting items for systematic reviews and meta-analyses (PRISMA) guidelines.^20^ The protocol was preregistered at the Prospective Register of Systematic Reviews (PROSPERO; CRD420251068370).^21^

### Search strategy

We conducted a comprehensive literature search in PubMed, Cochrane Library, Web of Science, and Embase, encompassing all records available until April 2025 without any language limitation. Additionally, we performed manual searches and citation tracking. Our main search keywords and related MeSH terms were ‘heart failure’, ‘SGLT-2 inhibitors’, and ‘sodium-glucose transporter 2’. Details of the search strategy can be found in the Supplementary material.

### Eligibility criteria

The following criteria determined eligibility for inclusion: randomized controlled trials (RCTs) enrolling adult participants (≥18 years old) diagnosed with any classification of HF; randomized to SGLT2 inhibitors versus non–SGLT2 inhibitor–based therapy, including placebo or active comparators; reporting mean, standard deviation (SD), and sample size for Hb and Hct at baseline and post-intervention in both groups, with a follow-up duration of at least 1 month. We excluded observational studies, crossover designs, uncontrolled trials, studies in which the entire population had anaemia at baseline and received treatments targeting anaemia, and studies that did not report the outcomes of interest or baseline characteristics.

### Study selection and data extraction

All eligible studies were imported into the Rayyan systematic review platform.^22^ After detecting and removing duplicates, two independent authors reviewed the titles and abstracts for eligibility (K.S. and A.S.). Next, the full texts of all included studies were independently evaluated by the same reviewers based on established inclusion criteria. Disagreements were resolved through discussion or consultation with a senior researcher (S.A.M.) when necessary. If two or more articles existed for a single trial, priority for choosing one of these articles was given to those with longer follow-up periods and more comprehensive outcome measures. Three independent authors (K.S., S.Z., and A.S.) extracted the study details using a predesigned Google sheet. For each included study, the following data were collected: first author, publication year, study design, baseline characteristics, types and dose of SGLT2 inhibitors and control groups, follow-up duration, mean, SD, and sample size of Hb and Hct levels from baseline. We resolved discrepancies through discussion with the senior author (S.A.M.).

### Quality assessment

Using the Cochrane Risk of Bias 2.0 (RoB-2) instrument, three reviewers (K.S., S.Z., and A.S.) independently evaluated the risk of bias in all included RCTs.^23^ Five principal domains are evaluated using this tool: the randomization process, any deviations from intended interventions, data on missing outcomes, measurement of outcomes, and selection of reported results. The judgements were categorized as ‘low risk of bias’, ‘some concerns’, and ‘high risk of bias’. We resolved discrepancies through consultation with the senior author (S.A.M.).

### Statistical analysis

Primary and secondary outcomes were changes in Hb and Hct levels from baseline to post-treatment follow-up. The mean differences (MDs) with 95% confidence intervals (CIs) between individual SGLT2 inhibitors and control groups were calculated using a random-effects model.^24^ SDs have been computed using the related guidelines, except where explicitly reported.^25^ For studies reporting median and range values, the mean and SD were estimated using appropriate formulas.^26^ We examined statistical heterogeneity with the χ^2^test (Cochran’s Q) and quantified it using the *I*^2^ statistic. Values above 50% were generally considered to indicate notable inconsistency.^27^

Subgroup analyses were performed across different SGLT2 inhibitors, follow-up durations, and types of control groups (placebo vs active comparators). Meta-regression analyses were conducted to assess the effect of baseline Hb and Hct, age, gender, body mass index (BMI), creatinine, DM, left ventricular ejection fraction (LVEF), follow-up time, estimated glomerular filtration rate (eGFR), heart rate (HR), hypertension (HTN), angiotensin-converting enzyme (ACE) inhibitors or angiotensin receptor blocker (ARB) use, angiotensin receptor neprilysin inhibitor (ARNI) use, Diuretics use on Hb and Hct changes. Funnel plots and Egger’s tests were used to explore publication bias.^28^ Statistical tests were two-sided, and a *P*-value < .05 was considered statistically significant. We used R version 4.2.3. (The R Foundation for Statistical Computing, Vienna, Austria) to conduct all analyses.^29^

## Results

**Figure 1:**
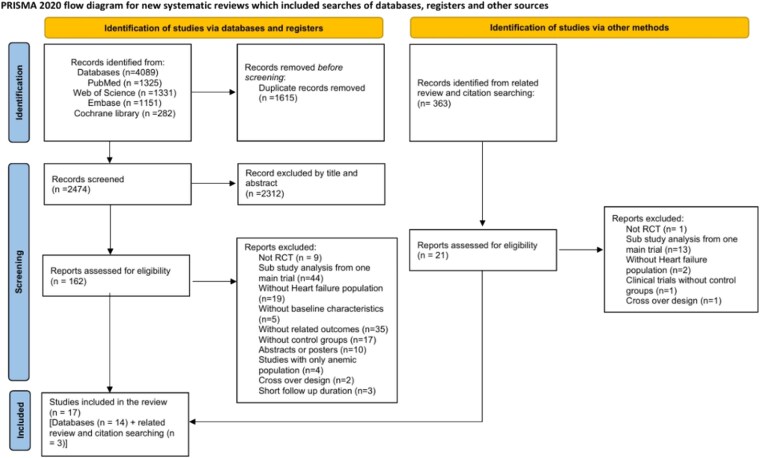
Preferred reporting items for systematic reviews and meta-analyses flow diagram of study selection; flow diagram showing the number of records identified, screened, excluded, and included in the systematic review and meta-analysis, in accordance with preferred reporting items for systematic reviews and meta-analyses 2020 guidelines

A total of 4089 records were retrieved through database searches, with an additional 363 records identified from other sources. After removing 1615 duplicates, 2474 records underwent title and abstract screening. Among these records, 162 full-texts were assessed for eligibility, and finally, 17 studies met our inclusion criteria. *Figure 1* (PRISMA flow chart) summarizes the study selection process.

### Study characteristics

Our study included 16 784 subjects, with an average age of 68.6 years, predominantly male (65.5%), and a mean follow-up duration of 18.2 months. The geographic distribution of the published articles included the UK (*n* = 1),^30^ USA (*n* = 3),^31–33^ multinational settings (*n* = 5),^34–38^ Denmark (*n* = 1),^39^ Scotland (*n* = 1),^40^ Greece (*n* = 1),^41^ Japan (*n* = 1),^42^ Korea (*n* = 1),^43^ Singapore (*n* = 1),^44^ Taiwan (*n* = 1),^45^ and Canada (*n* = 1).^46^ A detailed overview of study characteristics and participating countries is provided in *Table 1*.

**Table 1:** Baseline characteristics of included studies (HF, heart failure; T2DM, type 2 diabetes mellitus; HFpEF, heart failure preserved ejection fraction; SOC, standard of care; NYHA, New York Heart Association; EF, ejection fraction; HFrEF, heart failure reduced ejection fraction; LVEF, left ventricular ejection fraction; NT-proBNP, N-terminal pro–B-type natriuretic peptide)

Study characteristics								Case groups			Control groups			Outcomes
Author (Year)	Country	Trial name	Type of study	Study population	Follow-up Duration	Intervention	Comparison	*N*	Male%	Age	*N*	Male %	Age	
Lytvyn (2025) ^46^	Canada		Multi-centre, randomized, double-blind, placebo-controlled	HF (NYHA II class or III), EF ≥ 20% and T2DM	3 months	Ertugliflozin 15 mg daily	Placebo-controlled	17	88	69.8 ± 8.7	17	76	69.4 ± 7.5	Haemoglobin, Haematocrit
Lin (2025)^45^	Taiwan	ELUCIDATE trial	Single-centre, randomized, open-label, placebo-controlled	HFpEF (EF ≥ 50%) and asymptomatic T2DM	6 months	Dapagliflozin 10 mg daily	Active-controlled	38	45	54.3 ± 11.05	38	40	58.5 ± 9.02	Haemoglobin, Haematocrit
Tada (2024)^31^	USA	CAMEO-DAPA trial	Single-centre, randomized, double-blind, placebo-controlled	HFpEF (NYHA class II or III) and EF ≥ 50%	6 months	Dapagliflozin 10 mg daily	Placebo-controlled	21	33	67 ± 9	16	37	68 ± 9	Haematocrit
Marton (2024)^44^	Singapore	Dapa-shuttle trial	Single-centre, randomized, double-blind, placebo-controlled	Chronic HF (NYHA class I and II), and reduced EF	1 months	Dapagliflozin 10 mg daily	Placebo-controlled	15	80	55.8 ± 15.7	14	100	62.9 ± 10.5	Haematocrit
Kang (2024)^43^	Korea	EFFORT trial	Multi-centre, randomized, double-blind, placebo-controlled	HF (NYHA class II or III) and 35% ≤EF < 50%	12 months	Ertugliflozin	Placebo-controlled	63	63.5	65.4 ± 11.5	65	58.5	67.3 ± 11	Haemoglobin
Nakatani (2023)^42^	JAPAN	CANDLE trial	Multi-centre, randomized, open-label, blinded endpoint	HF (NYHA class I–III) and T2DM	6 months	Canagliflozin 10 mg daily	Active-controlled	109	78	68.6 ± 9.4	117	72.6	69.1 ± 10.3	Haemoglobin, Haematocrit
Katsiadas (2022)^41^	Greece		Single-centre, randomized, open-label, placebo-controlled	HF and T2DM	12 months	Dapagliflozin 10 mg daily	Active-controlled	56	83.9	68.1 ± 9.25	54	79.6	71.87 ± 9.1	Haemoglobin, Haematocrit
Voors (2022)^34^	Multinational	EMPULSE trial	Multi-centre, randomized, double-blind, placebo-controlled	Acute or decompensated chronic HF regardless of LVEF	3 months	Empagliflozin 10 mg daily	Placebo-controlled	265	67.5	70.3 ± 11.9	265	64.9	69 ± 14.2	Haemoglobin, Haematocrit
Nassif (2021)^32^	USA	EMBRACE-HF trial	Multi-centre, randomized, double-blind, placebo-controlled	HF (regardless of EF) with or without T2DM	3 months	Empagliflozin 10 mg daily	Placebo-controlled	33	63.6	69.5 ± 12	32	62.5	62.9 ± 13.3	Haemoglobin
Packer (2021)^35^	Multinational	Emperor-Preserved trial	Multi-centre, randomized, double-blind, placebo-controlled	Chronic HF (NYHA II–IV) and LVEF >40%	43 months	Empagliflozin 10 mg daily	Placebo-controlled	2997	49.7	71.9 ± 9.5	2991	48.9	72.2 ± 9.3	Haematocrit
Packer (2020)^36^	Multinational	Emperor-Reduced trial	Multi-centre, randomized, double-blind, placebo-controlled	Chronic HF (NYHA class II, III, or IV) and LVEF ≤40%	16 months	Empagliflozin 10 mg daily	Placebo-controlled	1863	76.5	67.2 ± 10.8	1867	75.6	66.5 ± 11.2	Haematocrit
Lee (2020)^40^	Scotland	SUGAR-DM-HF trial	Multi-centre, randomized, double-blind placebo-controlled	HF (NYHA class II to IV), LVEF ≤ 40% and T2DM or prediabetes	9 months	Empagliflozin 10 mg daily	Placebo-controlled	52	65.4	68.2 ± 11.7	53	81.1	69.2 ± 10.6	Haematocrit
Boer (2020)^37^	Multinational		Multi-centre, randomized double-blind, placebo-controlled	Symptomatic chronic HF (NYHA class II–IV) and T2DM	3 months	Licogliflozin 10 mg daily Empagliflozin 25 mg daily	Placebo-controlled	Lico: 16 Empa: 30	Lico: 25 Empa: 66.7	Lico: 71.3 ± 7.7 Empa: 68.2 ± 9.3	33	57.6	68 ± 11.62	Haematocrit
Gallego (2020)^33^	USA	EMPATROPIM trial	Single-centre, randomized, double-blind, placebo-controlled	HFrEF (NYHA class II-III), EF < 50% without diabetes	6 months	Empagliflozin	Placebo-controlled	42	63	64.2 ± 10.9	42	64	59.9 ± 13.1	Haemoglobin, Haematocrit
Omar (2020)^39^	Denmark	EMPIRE trial	Multi-centre, randomized, double-blind, placebo-controlled	HFrEF (NYHA class I–III) and EF ≤ 40%	3 months	Empagliflozin 10 mg daily	Placebo-controlled	95	83	65 ± 10	95	87	63 ± 12	Haematocrit
McMurray (2019)^30^	U. K	DAPA-HF trial	Multi-centre, randomized, double-blind, placebo-controlled	HF (NYHA class II, III, or IV) and EF ≤ 40%	18.2 months	Dapagliflozin 10 mg daily	Placebo-controlled	2373	76.2	66.2 ± 11	2371	77	66.5 ± 10.8	Haematocrit
Kosiborod (2017)^38^	Multinational		Post-hoc pooled analysis of data from five multi-centre, randomized, placebo-controlled trials	HF (NYHA class I−III) and T2DM	13 months	Dapagliflozin 10 mg daily	Placebo-controlled	171	64.3	63.6 ± 7.5	149	61.1	64.9 ± 7.3	Haemoglobin, Haematocrit

### Quality assessment

We assessed the reliability of all studies using the RoB 2 tool. Except for three studies judged as having a high risk of bias^37,41,42^ primarily due to issues in maintaining the intended intervention, the remaining studies were rated as either ‘low risk’^30–32,34–36,39,40,43–46^ or ‘some concerns’.^33,38^ This suggests that the comparison between SGLT2 inhibitors and the control group in HF patients is likely reliable. A summary of the quality assessment across all domains is provided in Supplementary Figs S1 and S2.

### Effects of sodium-glucose cotransporter-2 inhibitors on haemoglobin (g/dl) and haematocrit (%)

#### Haemoglobin (g/dl)

Nine of the included RCTs were eligible for inclusion in the meta-analysis of Hb^32–34,38,41–43,45,46^ (*n* = 1465). Treatment with SGLT2 inhibitors was associated with a significant increase in Hb levels compared with the control group (MD = 0.68 g/dl, 95% CI: 0.53; 0.83, *P*-value < .0001).

**Figure 2:**
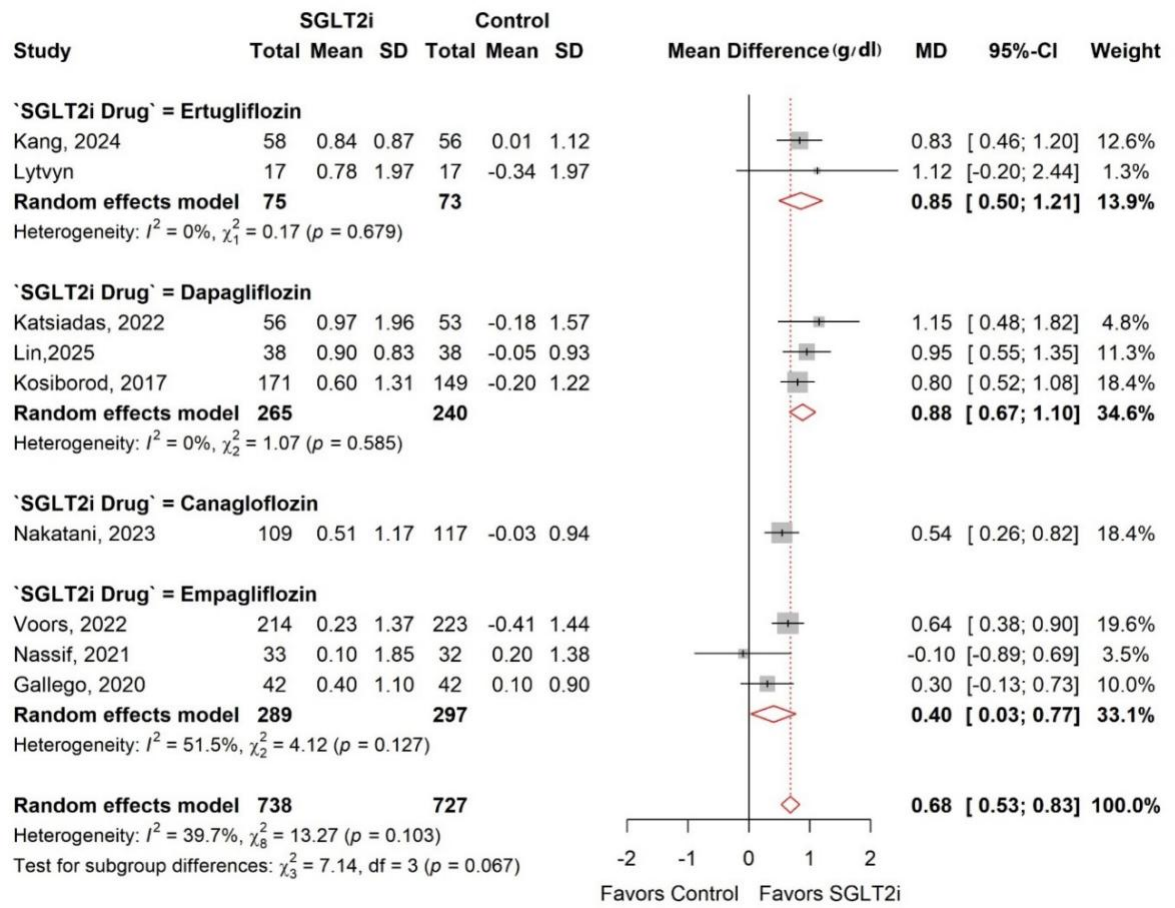
Meta-analysis of mean difference and 95% confidence interval of changes in haemoglobin levels (Hb, g/dl) across all sodium-glucose cotransporter-2 inhibitors drug groups. Mean differences are from a random-effects model analysis. CI, confidence interval; MD, mean difference

Subgroup analyses showed consistent benefits across all SGLT2 inhibitors classes, with the largest effects observed for dapagliflozin (MD = 0.88 g/dl, 95% CI: 0.67; 1.10) and ertugliflozin (MD = 0.85 g/dl, 95% CI: 0.50; 1.21), while empagliflozin while empagliflozin demonstrated smallest but still statistically significant effect (MD = 0.40 g/dl, 95% CI: 0.03; 0.77). Between-study heterogeneity was moderate (*I*^2^ = 39.7%), and we found no significant differences between drug subgroups (*P* = .067), indicating a consistent treatment effect across the drug classes (*Figure 2*).

**Figure 3:**
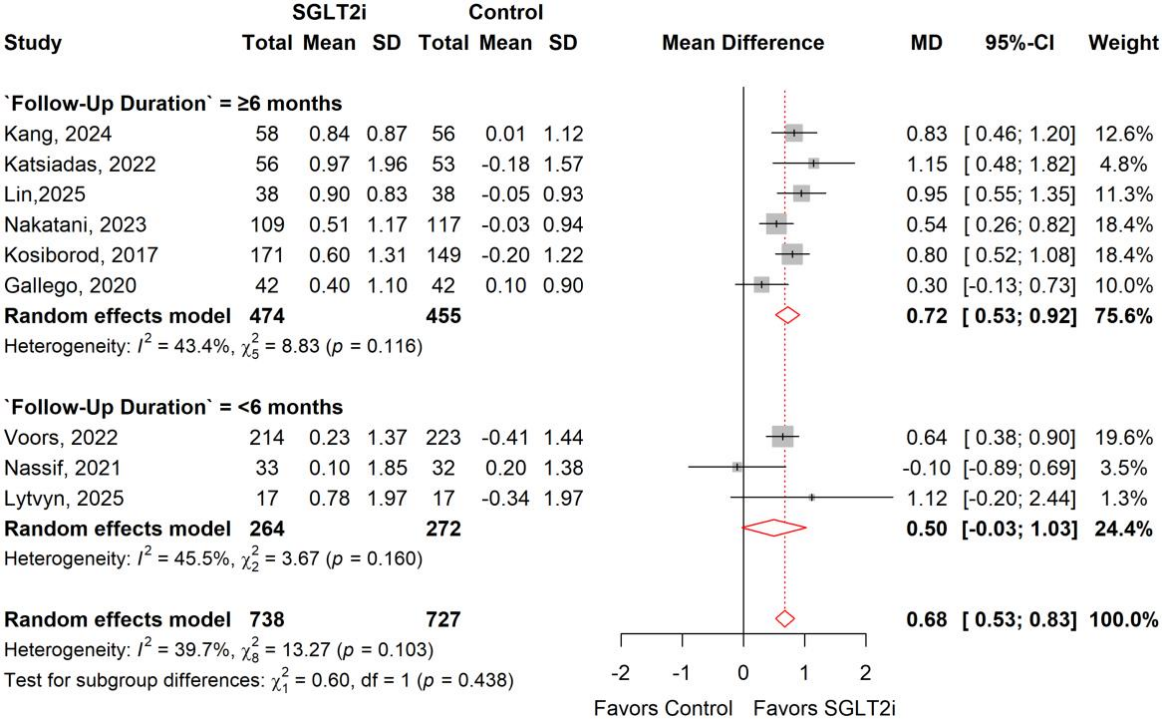
Subgroup analysis of sodium-glucose cotransporter-2 inhibitors on haemoglobin (g/dl) changes stratified by follow-up duration (<6 months vs ≥6 months): meta-analysis of mean difference (g/dl) and 95% confidence interval across all drug groups using a random-effects model. CI, confidence interval; MD, mean difference

Additional pre-specified subgroup analyses were conducted by follow-up duration and control group types. Studies with a follow-up of at least 6 months demonstrated a greater increase in Hb levels with SGLT2 inhibitor therapy (≥6 months; MD = 0.72 g/dl, 95% CI: 0.53; 0.92) compared with studies with shorter follow-up (<6 months; MD = 0.50 g/dl, 95% CI: − 0.03; 1.03), however the difference between subgroups was not statistically significant (*P* = .438) (*Figure 3*).

When the analyses were stratified by control groups, both placebo-controlled trials (MD = 0.63 g/dl, 95% CI: 0.43; 0.84) and active-controlled trials (MD = 0.81 g/dl, 95% CI: 0.44; 1.17) showed significant increase in Hb levels associated with SGLT2 inhibitor use, with no evidence of effect modification by control type (*P* = .416) (Supplementary Fig. S3).

#### Haematocrit (%)

**Figure 4:**
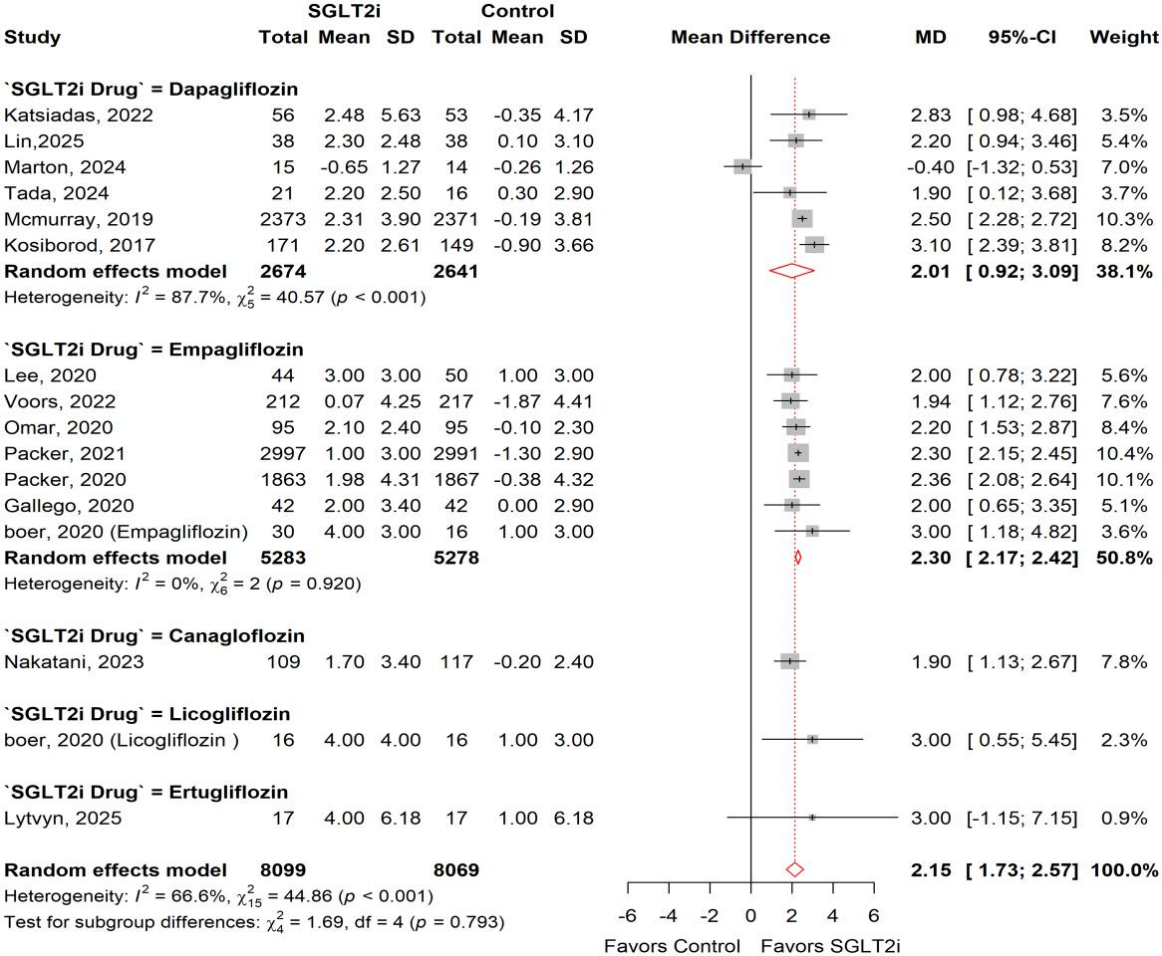
Meta-analysis of mean difference and 95% confidence interval of changes in haematocrit levels (%) across all sodium-glucose cotransporter-2 inhibitors drug groups. Mean differences are from a random-effects model analysis. CI, confidence interval; MD, mean difference

Among the 17 studies included in this study, 15 RCTs were eligible for inclusion in the meta-analysis of Hct.^30,31,33–42,44–46^ As shown in *Figure 4*, treatment with SGLT2 inhibitors was significantly associated with higher Hct levels compared to the control group (MD = 2.15%, 95% CI: 1.73; 2.57, *P*-value < .0001). Substantial heterogeneity was observed between studies (*I*^2^ = 66.6%).

Subgroup analysis by drug type showed no significant differences between SGLT2 inhibitor classes (χ^2^ = 1.69, df = 4, *P* = .793). Dapagliflozin (MD = 2.01%, 95% CI: 0.92; 3.09), empagliflozin (MD = 2.30%, 95% CI: 2.17; 2.42), canagliflozin (MD = 1.90%, 95% CI: 1.13; 2.67), and licogliflozin (MD = 3%, 95% CI: 0.55; 5.45) all showed a significant increase in Hct levels. However, the effect of ertugliflozin (MD = 3%, 95% CI: –1.15; 7.15) was not statistically significant. No statistically significant differences were detected between drug subgroups (*P* = .793), supporting a class effect (*Figure 4*).

**Figure 5:**
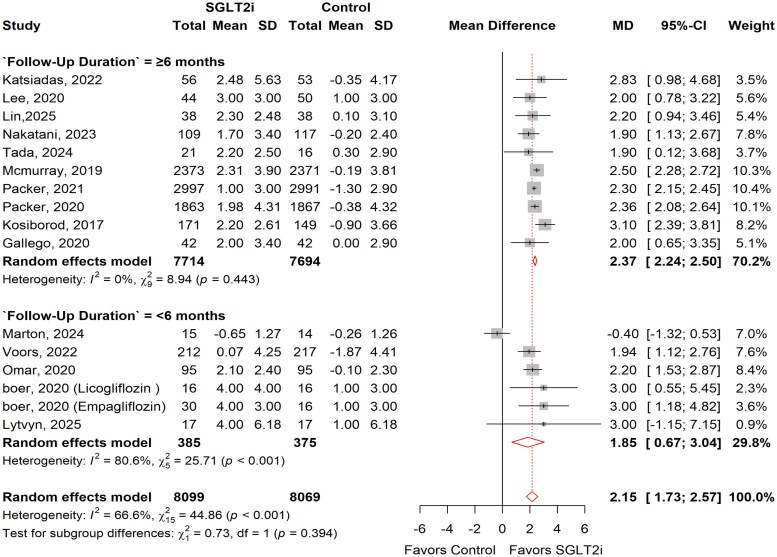
Subgroup analysis of sodium-glucose cotransporter-2 inhibitors on haematocrit (%) changes stratified by follow-up duration (<6 months vs ≥6 months): meta-analysis of mean difference (%) and 95% confidence interval across all drug groups using a random-effects model. CI, confidence interval; MD, mean difference

In subgroup analysis stratified by follow-up duration, studies with ≥ 6 months of follow-up showed a more pronounced increase in Hct (MD = 2.37%, 95% CI: 2.24; 2.50) compared with studies with <6 months (MD = 1.85%, 95% CI: 0.67; 3.04); however, the between-group difference was not significant (*P* = .394) (*Figure 5*). Similarly, both placebo-controlled (MD = 2.14%, 95% CI: 1.63; 2.65) and active-controlled trials (MD = 2.08%, 95% CI: 1.46; 2.70) demonstrated significant increases in Hct with SGLT2 inhibitor therapy, with no evidence of effect modification by control type (*P* = .881) (Supplementary Fig. S4). These findings indicate a consistent increase in Hct across SGLT2 inhibitor agents, treatment durations, and comparator groups, reinforcing a reproducible haematologic effect in patients with HF.

### Meta-regression

Meta-regression analyses showed a significant inverse association between baseline Hct and the MD in Hct change with SGLT2 inhibitor therapy compared with control (regression coefficient [β]: −0.56 per 1% increase in baseline Hct; *P* < .0001; Supplementary Fig. S5). The analysis indicated that lower baseline Hct was associated with larger increases in Hct. However, the modifying effect of baseline Hct was modest in magnitude. The corresponding bubble plot illustrating this association is shown in Supplementary Fig. S5.

There were no significant associations between the Hb- and Hct-increasing effects of SGLT2 inhibitors and other pre-specified study-level covariates, including age, proportion of male participants, diuretic use, HTN, DM, baseline creatinine, eGFR, BMI, LVEF, use of ACE inhibitors or ARB, use of ARNI, HR, or follow-up duration (all *P* > .05). Results of the meta-regression for all potential moderators are presented in Supplementary Figs. S5 and S6.

### Publication bias

There was no significant publication bias for the effects of SGLT2 inhibitors in both Hb and Hct across studies, as indicated by symmetrical funnel plots, further confirmed by Egger's test (Hb. Egger *P* = .9690, Hct. Egger *P* = .4627) (Supplementary Figs S7 and S8).

## Discussion

This systematic review and meta-analysis of RCTs was performed to evaluate the effects of SGLT2 inhibitors on Hb and Hct levels in patients with HF. Our findings demonstrate that treatment with SGLT2 inhibitors is associated with a statistically significant but modest increase in both Hb and Hct levels in this population. Between-study heterogeneity was moderate for Hb (*I*^2^ ≈ 40%) and high for Hct (*I*^2^ ≈ 67%), reflecting variability in trial populations and designs. Subgroup analyses, including variations in drug classes, follow-up durations, and control groups, yielded consistent results, underscoring the robustness of our findings. Meta-regression analyses showed a significant inverse association between baseline Hct and the MD in Hct change with SGLT2 inhibitor therapy, whereas baseline Hb did not influence changes in Hb.

These results are consistent with prior reports in non–HF populations. Kanbay *et al*. demonstrated a significant increase in Hb and Hct levels with SGLT2 inhibitors in Type 2 DM populations.^17^ Our analysis extends these observations specifically to patients with HF, a population in which anaemia is common and prognostically important.

Previous meta-analyses consistently highlight the cardiovascular and renal protective effects of SGLT2 inhibitors.^12,47,48^ However, the impact of SGLT2 inhibitors on haematopoietic parameters has been specifically less explored, with most investigations evaluating Hb and Hct changes conducted in populations with DM or CKD. For instance, Luo *et al*. demonstrated that treatment with SGLT2 inhibitors was associated with significant increases in both Hb and Hct levels.^16^ Similarly, another study conducted in populations with concomitant DM and CKD has reported consistent improvements in haematologic parameters, showing that SGLT2 inhibitors increase Hb levels in these high-risk patients.^49^

Anaemia is a common and clinically significant comorbidity in patients with HF, arising from a combination of iron deficiency, chronic inflammation, renal dysfunction, and impaired erythropoiesis.^5^ The presence of anaemia in HF has been consistently associated with increased morbidity, higher rates of hospitalization, and excess mortality.^50^ Despite its clinical importance, prior attempts to directly correct anaemia in HF have yielded inconsistent results.^7,51–53^ Large randomized trials of intravenous iron therapy (AFFIRM-AHF, IRONMAN, HEART-FID) have demonstrated improvements in symptoms and functional status, with inconsistent effects on HF hospitalizations and no clear or consistent benefit on cardiovascular mortality, highlighting the heterogeneity of therapeutic responses.^51–53^ Additionally, in the RED-HF trial, treatment of anaemic patients with systolic HF using the erythropoiesis-stimulating agent (darbepoetin alfa) failed to reduce the risk of death or hospitalization and was instead associated with a significantly increased incidence of thromboembolic events (RED-HF trial).^7^ This study concluded that Hb primarily functions as an indicator of poor prognosis in HF, rather than a modifiable treatment target.^7^

On the other hand, large cardiovascular outcome trials have consistently demonstrated that treatment with SGLT2 inhibitors significantly reduces the risk of hospitalization and cardiovascular mortality in patients with HF.^36,54^ Mediation analyses from these trials have further indicated that changes in Hb and Hct are among the strongest contributors to the observed reductions in HF hospitalization and death.^55^ In light of these findings, and given the established cardiovascular benefits of SGLT2 inhibitors, the present study aimed to specifically quantify changes in Hb and Hct in patients with HF receiving SGLT2 inhibitor therapy, without evaluating clinical outcomes. Accordingly, our analysis was not designed to assess HF– or cardiovascular-related endpoints, but rather focused exclusively on haematologic changes following SGLT2 inhibitor use as a prognostic predictor. Moreover, we excluded studies in which the entire study population had anaemia at baseline and received active anaemia-targeted therapies, such as iron supplementation or erythropoiesis-stimulating agents, to better delineate haematologic effects attributable to SGLT2 inhibitors and minimize confounding. Additionally, we performed meta-regression analyses to examine whether baseline use of ACE inhibitors, ARBs, ARNI, or diuretics influenced the association between SGLT2 inhibitor therapy and changes in Hb and Hct; no significant associations were observed.

Unlike erythropoiesis-stimulating agents and iron therapy, which directly target erythropoiesis and iron availability,^56,57^ the haematologic effects of SGLT2 inhibitors appear to arise from indirect, regulated, and multifactorial pathways.^58^ Although the initial rise in Hct following SGLT2i initiation has often been attributed to plasma volume contraction secondary to osmotic diuresis, this explanation alone is insufficient.^59^ Accordingly, the diuretic-induced increase in urine output peaks within 24 h and returns to baseline within a week, whereas Hct levels continue to rise for up to 2 months, indicating that the sustained elevation cannot be explained solely by transient hemoconcentration.^60,61^ Supporting this, Tian *et al*.^48^ demonstrated that SGLT2 inhibitors increase Hct and Hb levels in patients with Type2 DM, primarily through enhanced erythropoietin synthesis rather than simple plasma volume contraction from diuresis. These changes reflect improved renal metabolic stress and reduced sympathetic overactivity, suggesting that elevations in Hb and Hct may act as key mediators of the cardiorenal protective effects of SGLT2 inhibitors.^48^ Beyond haemoconcentration, it has been proposed that SGLT2 inhibitors may enhance erythropoiesis by restoring peritubular fibroblast function and stimulating erythropoietin production. By alleviating glucose reabsorption and metabolic stress in proximal tubular cells, they reverse hypoxia and fibrosis, leading to improved erythropoietin release,^62^ supported by trial data showing sustained elevations up to 12 weeks.^63,64^ These drugs also improve iron handling by lowering hepcidin and ferritin, increasing soluble transferrin receptors and total iron-binding capacity, thereby enhancing iron availability for red blood cell production.^65^ These mechanisms may also contribute to the sustained elevation in Hb and Hct observed with SGLT2 inhibitors. Notably, in our study, patients had eGFR values between 60 and 90 ml/min/1.73 m^2^. Although erythropoietin deficiency begins early in CKD, it becomes markedly more pronounced when eGFR falls below 30 ml/min/1.73 m^2^.^66^ Therefore, the observed increases in Hb and Hct in our study are likely attributable to increased erythropoiesis in addition to volume-related effects.

Importantly, our subgroup analysis further supports a biological rather than purely haemodynamic explanation, as studies with follow-up durations longer than 6 months demonstrated significantly greater increases in Hb compared with studies with shorter follow-up periods. This temporal pattern suggests a progressive and sustained effect consistent with enhanced erythropoiesis and improved iron metabolism, rather than short-term haemoconcentration, which would be expected to occur early and plateau over time.^63,67^ However, these proposed mechanisms should be interpreted with caution, as they remain biologically plausible but cannot be definitively confirmed because the trials included in our analysis did not evaluate relevant biomarkers such as erythropoietin, ferritin, hepcidin, or soluble transferrin receptors. Therefore, additional studies incorporating a detailed assessment of these biomarkers are required to better elucidate and confirm the mechanisms by which SGLT2 inhibitors exert their effects.

Further insight into the potential effect modifiers of Hct and Hb changes is provided by our meta-regression and subgroup analyses. In our meta-regression, baseline Hct, but not baseline Hb, was a significant moderator of the treatment effect. In subgroup analyses stratified by follow-up duration, studies with follow-up shorter than 6 months were not associated with significant changes in Hb, whereas they were associated with significant increases in Hct. Consistently, baseline Hct, but not baseline Hb, emerged as a significant moderator in meta-regression. These findings support the concept that early increases in Hct predominantly reflect short-term haemoconcentration and haemodynamic changes following SGLT2 inhibitor initiation,^68–70^ while also indicating that Hb changes emerge primarily over longer follow-up periods, consistent with sustained biological effects rather than acute volume shifts.^67^ In most modern laboratories, Hct is not measured directly but is calculated from the red blood cell (RBC) count and mean corpuscular volume, whereas Hb is measured directly.^71^ As a result, Hct is inherently more susceptible to variability, reflecting changes in both RBC number and cell size, and is therefore more sensitive to acute plasma volume contraction.^71,72^

In contrast, Hb more closely reflects absolute RBC mass and is less affected by transient volume changes,^63,73^ which may explain the absence of a significant association with baseline Hb in our analysis. Additionally, the larger number of studies reporting Hct outcomes compared with Hb may have increased statistical power, contributing to the observed significance for Hct. Future studies incorporating direct measurements of RBC mass and plasma volume are warranted to more precisely delineate these mechanisms.

Subgroup analyses by individual SGLT2 inhibitor showed no statistically significant MDs between drug classes, although numerical differences were observed among individual agents, which may be due to different study designs or study population, and also differences in the mechanism and pharmacology of the various SGLT2 inhibitors. The selectivity of SGLT2 inhibitors for SGLT2 over SGLT1 is an important pharmacologic factor that may influence haematologic parameters.^16^ Canagliflozin has the lowest SGLT2 selectivity, while empagliflozin has the highest.^74^ Consistent with this, Lou *et al.*^16^ reported a stronger effect of canagliflozin in patients with Type 2 DM in increasing Hct, likely due to its dual inhibition of both SGLT2 and SGLT1. Polidori *et al*.^75^ also showed that canagliflozin reduces postprandial glucose and insulin excursions not only via renal SGLT2-mediated urinary glucose excretion but also by delaying intestinal glucose absorption through SGLT1 inhibition. However, in our study, canagliflozin showed the smallest numerical increase in Hct, which may be explained by the inclusion of only a single study assessing this agent. Additionally, empagliflozin was assessed in seven studies and demonstrated a consistent effect on Hct, with a mean increase of 2.30% (95% CI: 2.17; 2.43) in the random-effects model. Notably, this subgroup showed no heterogeneity (*I*^2^ = 0%), indicating highly consistent results across studies. The narrow CI further reflects the precision of the estimate, suggesting that empagliflozin’s effect on Hct is both reliable and reproducible across different patient populations and study designs.

Regarding Hb, the pooled effects were generally consistent across ertugliflozin, dapagliflozin, and canagliflozin, with low heterogeneity (*I*^2^ = 0%), suggesting a class-wide hemoconcentration effect. However, empagliflozin showed moderate heterogeneity (*I*^2^ = 51%), which may reflect differences in patient populations, background therapy, or study design among the included trials.

While our meta-regression analysis did not demonstrate significant differences in Hb or Hct changes across the spectrum of LVEF, prior RCTs have shown that the cardiovascular benefits of SGLT2 inhibitors vary according to EF phenotype.^34–36^ This apparent dissociation suggests that the haematologic effects of SGLT2 inhibitors are likely mediated by systemic and renal mechanisms that are relatively independent of systolic function, whereas downstream cardiovascular outcomes may be more strongly influenced by EF-specific pathophysiology. Accordingly, further adequately powered studies stratified by EF are needed to clarify whether subtle differences in haematologic responses exist and to better understand how these changes translate into differential clinical outcomes across HF phenotypes.

### Study limitations

To our knowledge, this is the first systematic review and meta-analysis to specifically evaluate the effects of SGLT2 inhibitors on Hb and Hct levels in patients with HF. However, several limitations should be acknowledged.

First, changes in Hb and Hct were not predefined primary outcomes in the included trials, resulting in limited statistical power for these endpoints. Second, this was a study-level meta-analysis based on aggregate data; individual participant data were not available, so clinically relevant outcomes such as the proportion of patients achieving a ≥ 1 g/dl increase in Hb or a ≥ 1% increase in Hct could not be assessed. In addition, differences in inclusion and exclusion criteria across trials limited adjustment for patient-level characteristics. Third, patients with baseline anaemia were excluded to avoid the influence of concomitant anaemia-related therapies that could affect haematologic parameters, which limits the applicability of our findings to this subgroup; therefore, the impact of SGLT2 inhibitors on individuals with pre-existing anaemia should be assessed in future studies. Fourth, the majority of included patients had preserved renal function (eGFR > 60 ml/min/1.73 m^2^), so the impact on patients with renal anaemia remains unclear. In addition, for drugs assessed by only one study, the evidence is particularly limited and requires validation in larger populations. Finally, moderate to substantial heterogeneity—particularly for Hct—was observed and largely remained unexplained despite the use of random-effects models, subgroup analyses, and meta-regression, likely reflecting the use of aggregate data and the multifactorial nature of HF populations; therefore, the results should be interpreted with caution.

## Conclusion

In this systematic review and meta-analysis of RCTs, treatment with SGLT2 inhibitors was associated with a statistically significant increase in both Hb and Hct levels in patients with HF. However, the mechanisms and clinical significance of these haematologic changes are not yet fully understood. Additional prospective studies with integrated biomarker evaluation of erythropoiesis, iron metabolism, and plasma volume, and inclusion of patient-level data across a wider range of renal function and anaemia status, are warranted.

## Supplementary Material

xvag027_Supplementary_Data

## Supplementary data

Use of AI and AI-assisted technologies statement: During the preparation of this work the authors used ChatGPT 4o, before having English proofread by an expert in order to English sentence correction. After using this service, the authors reviewed and edited the content as needed and take full responsibility for the content of the publication. Disclosure of AI Programs We employed AI tools, notably ChatGPT 5.2 and Grammarly GO, to assist in rephrasing segments of this article. The objective was solely to enhance clarity and readability. Nevertheless, all AI generated content was meticulously reviewed and verified by the authors to guarantee accuracy and integrity.

## Declarations

### Data Availability

Availability of data and materials: The corresponding author will provide further information upon reasonable request due to confidentiality/privacy.
