# Supplementary material for: Effect of sodium-glucose cotransporter-2 inhibitors on haemoglobin and haematocrit levels in heart failure: a systematic review and meta-analysis
Source: ESC Heart Fail. 2026 Jan 19;13(3):xvag027. doi: 10.1093/eschf/xvag027 (PMC13262745; doi:10.1093/eschf/xvag027)

**Effect of SGLT2 inhibitors on Hemoglobin and Hematocrit Level in Patients with Heart Failure: a systematic review and meta-analysis**

Supplemental Material

Table of Contents

[Search Strategy 2](#_Toc180508952)

[PubMed 2](#_Toc180508953)

[Cochrane Library 3](#_Toc180508954)

[Embase 4](#_Toc180508955)

[Web of Science 4](#_Toc180508956)

[Total Results 4](#_Toc180508957)

[Quality Assessment 5](#_Toc180508958)

[Supplemental Figure 1: The summary of risk of bias assessment using the Cochrane Risk of Bias 2.0 (RoB-2) tool 5](#_Toc180508959)

[Supplemental Figure 2: Risk of bias assessed using ROB-2; visualization with Robovis (weighted summary across six domains. 6](#_Toc180508960)

[Supplemental Results 7](#_Toc180508961)

[Supplemental Figure 3: Subgroup analysis of SGLT2 inhibitors on hemoglobin (Hb) changes by control group (placebo-controlled vs active-controlled): Meta-analysis of mean difference (MD) and 95% confidence intervals (CIs) across all drug groups using a random-effects model 7](#_Toc180508962)

[Supplemental Figure 4: Subgroup analysis of SGLT2 inhibitors on Hematocrit (Hct) changes by control group (placebo-controlled vs active-controlled): Meta-analysis of mean difference (MD) and 95% confidence intervals (CIs) across all drug groups using a random-effects model 8](#_Toc180508964)

[Supplemental Figure 5: Meta-regression analysis examining the association between baseline hematocrit (Hct), Age, Body Mass Index (BMI), Creatinine (Cr), Diabetes Mellitus (DM), Hypertension (HTN), estimated Glomerular Filtration Rate (eGFR), Follow-up duration, Heart Rate (HR), Male sex and hematocrit (Hct) response to SGLT2 inhibitors therapy](#_Toc180508966) 9-10-11

[Supplemental Figure 6: Meta-regression analysis examining the association between baseline hemoglobin (Hb), Age, Body Mass Index (BMI), Creatinine (Cr), Diabetes Mellitus (DM), Hypertension (HTN), estimated Glomerular Filtration Rate (eGFR), Follow-up duration, Heart Rate (HR), Male sex, Ejection Fraction (EF), Angiotansin-converting enzyme (ACE) inhibitors or Angiotansin Receptor Blocker (ARB) use, Angiotansin Receptor Neprilysin Inhibitor (ARNI) use, Diuretics use and Hb response to SGLT2 inhibitors therapy](#_Toc180508967) 12-13-14

[Supplemental Figure 7: Funnel plot of hemoglobin (Hb) 14](#_Toc180508967)

[Supplemental Figure 8: Funnel plot of hematocrit (Hct) 15](#_Toc180508967)

# Search Strategy

---------------------------------------------------------------------------------------------------------------------

**PubMed**

**Search date: 20/4/2025**

**String #1:**

"Sodium-Glucose Transporter 2 Inhibitors"[Mesh] OR "sodium*glucose transporter 2 inhibitor*"[Title/Abstract] OR "sglt2 inhibitor*"[Title/Abstract] OR “sglt*2 antagonist*" [Title/Abstract] OR "sotagliflozin"[Title/Abstract] OR "empagliflozin"[Supplementary Concept] OR "empagliflozin"[Title/Abstract] OR "canagliflozin"[Mesh] OR "canagliflozin"[Title/Abstract] OR "dapagliflozin"[Title/Abstract]

**String #2:**

("heart failure"[Mesh] OR ("heart failure"[Title/Abstract]) OR (("cardiac"[Title/Abstract] AND "failure"[Title/Abstract]) OR "cardiac failure"[Title/Abstract]) OR "Heart Failure, Diastolic"[Mesh] OR "Heart Failure, Systolic"[Mesh] OR "heart failure with reduced ejection fraction"[Title/Abstract] OR "heart failure and reduced ejection fraction"[Title/Abstract] OR "HFrEF"[Title/Abstract] OR "heart failure with preserved ejection fraction"[Title/Abstract] OR "heart failure and preserved ejection fraction"[Title/Abstract] OR "HFpEF"[Title/Abstract])

**String #3:**

(Random*)

**Syntax:**

#1 AND #2 AND #3

**Number of results: 1325**

**Cochrane library**

**String #1:** MeSH descriptor: [Sodium-Glucose Transporter 2 Inhibitors] explode all trees

**String #2:** ("sodium*glucose transporter 2 inhibitor*" OR "sglt*2 inhibitor*" OR "sglt*2 antagonist*")

**String #3:** #1 AND #2

**String #4**: MeSH descriptor: [Heart Failure] explode all trees

**String #5**: MeSH descriptor: [Heart Failure, Diastolic] explode all trees

**String #6**: MeSH descriptor: [Heart Failure, Systolic] explode all trees

**String #7**: #5 OR #6 OR #7

Syntax: #3 AND #7

**Number of results: 282**

**EmBase**

**String #1:**

'sodium*glucose transporter 2 inhibitor*':ti,ab OR 'sglt*2 inhibitor*':ti,ab OR 'sglt*2 antagonist*':ti,ab OR 'sodium glucose cotransporter 2 inhibitor'/exp

**String #2:**

'heart failure with reduced ejection fraction'/exp OR 'heart failure with preserved ejection fraction'/exp

**String #3:**

random*

**Syntax:**

#1 AND #2 AND #3

**Number of results: 1151**

**Web of science**

("Sodium-Glucose Transporter 2 Inhibitors" OR "sodium-glucose transporter 2 inhibitor*" OR "SGLT2 inhibitor*" OR "SGLT2 antagonist*" OR "sotagliflozin" OR "empagliflozin" OR "empagliflozin" OR "canagliflozin" OR "canagliflozin" OR "dapagliflozin") AND ("heart failure" OR "heart failure" OR "cardiac failure" OR "Heart Failure, Diastolic" OR "Heart Failure, Systolic" OR "heart failure with reduced ejection fraction" OR HFrEF OR "heart failure with preserved ejection fraction" OR "HFpEF") AND ("Random*")

**Number of results: 1331**

## Total Results

| Data Base | Results (No.) |
| --- | --- |
| PubMed | 1325 |
| Cochrane Library | 282 |
| Embase | 1151 |
| WOS | 1331 |
| Total | **4089** |

#

# Quality Assessment

---------------------------------------------------------------------------------------------------------------------

Supplemental Figure S1: The summary of risk of bias assessment using the ROB-2 tool

**
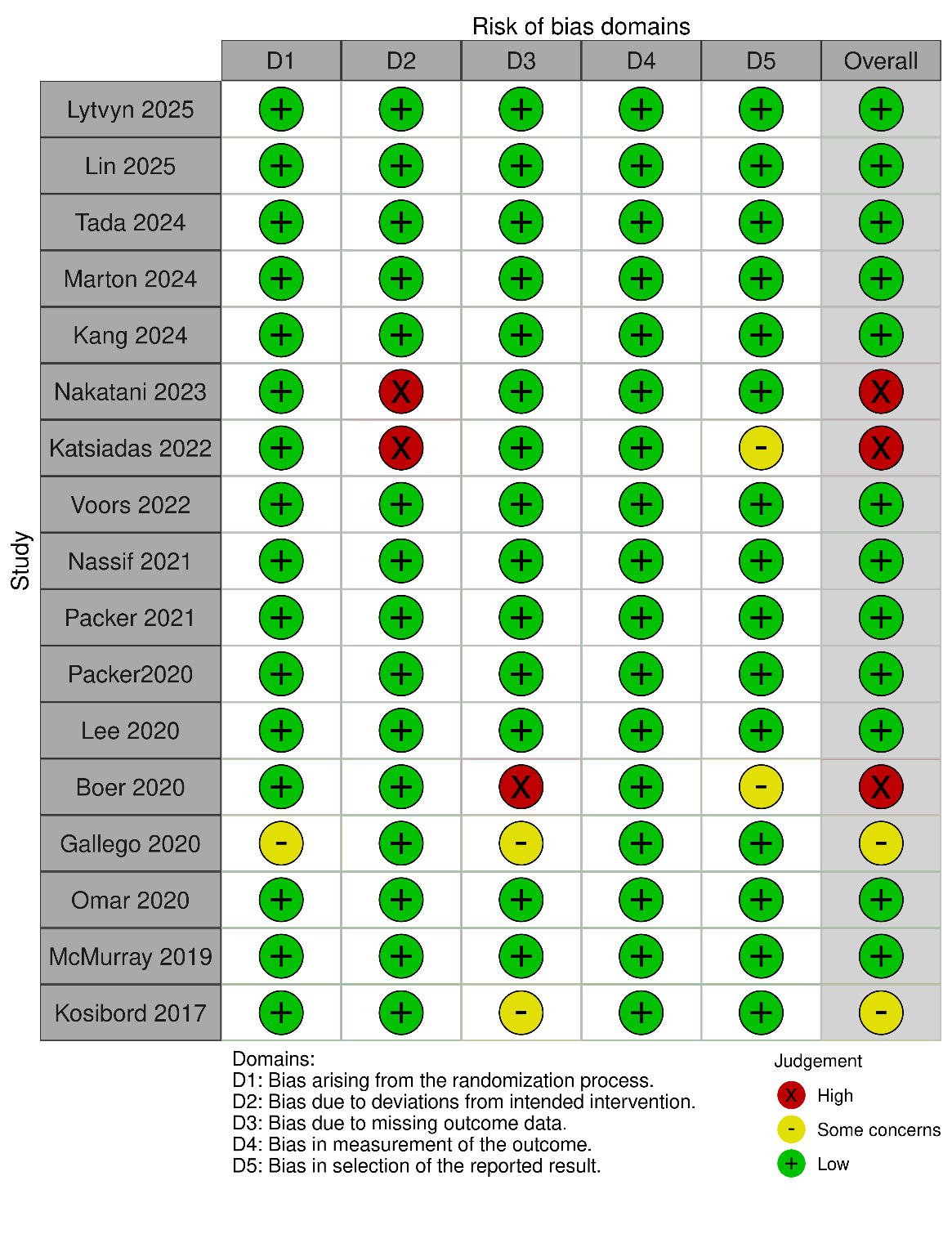
**

Supplemental Figure S2: Risk of bias assessed using ROB-2 tool applied utilizing Robovis online software to visualize weighted summary plot across six risk of bias domains.

**
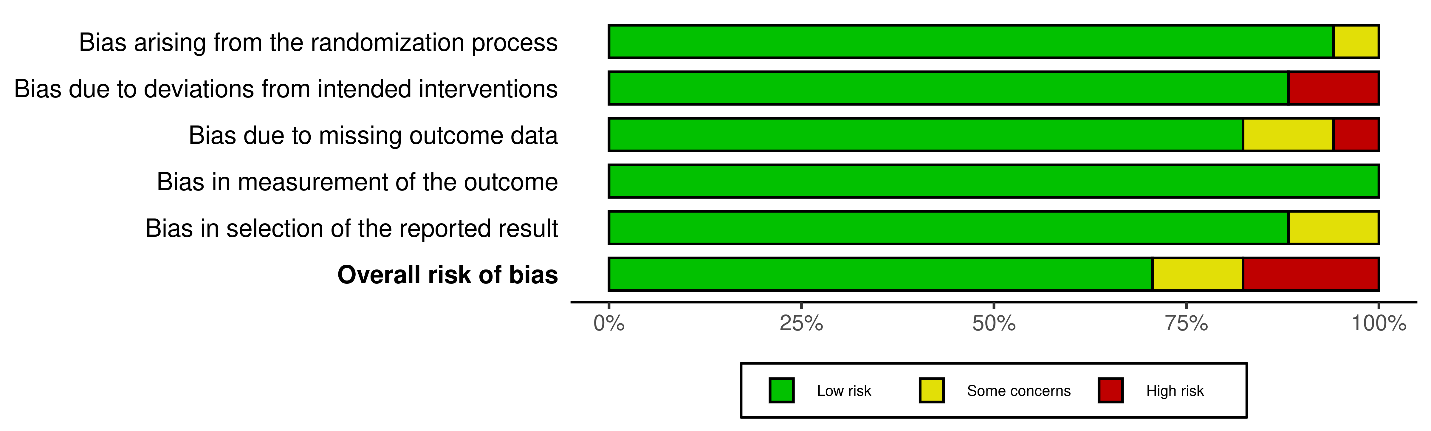
**

#

# Supplemental Results

---------------------------------------------------------------------------------------------------------------------

## Supplemental Figure S3: Subgroup analysis of SGLT2 inhibitors on hemoglobin (Hb, g/dL) changes by control group (placebo-controlled vs active-controlled): Meta-analysis of MD and 95% confidence intervals (CIs) across all drug groups using a random-effects model. CI: confidence interval; MD: mean difference.


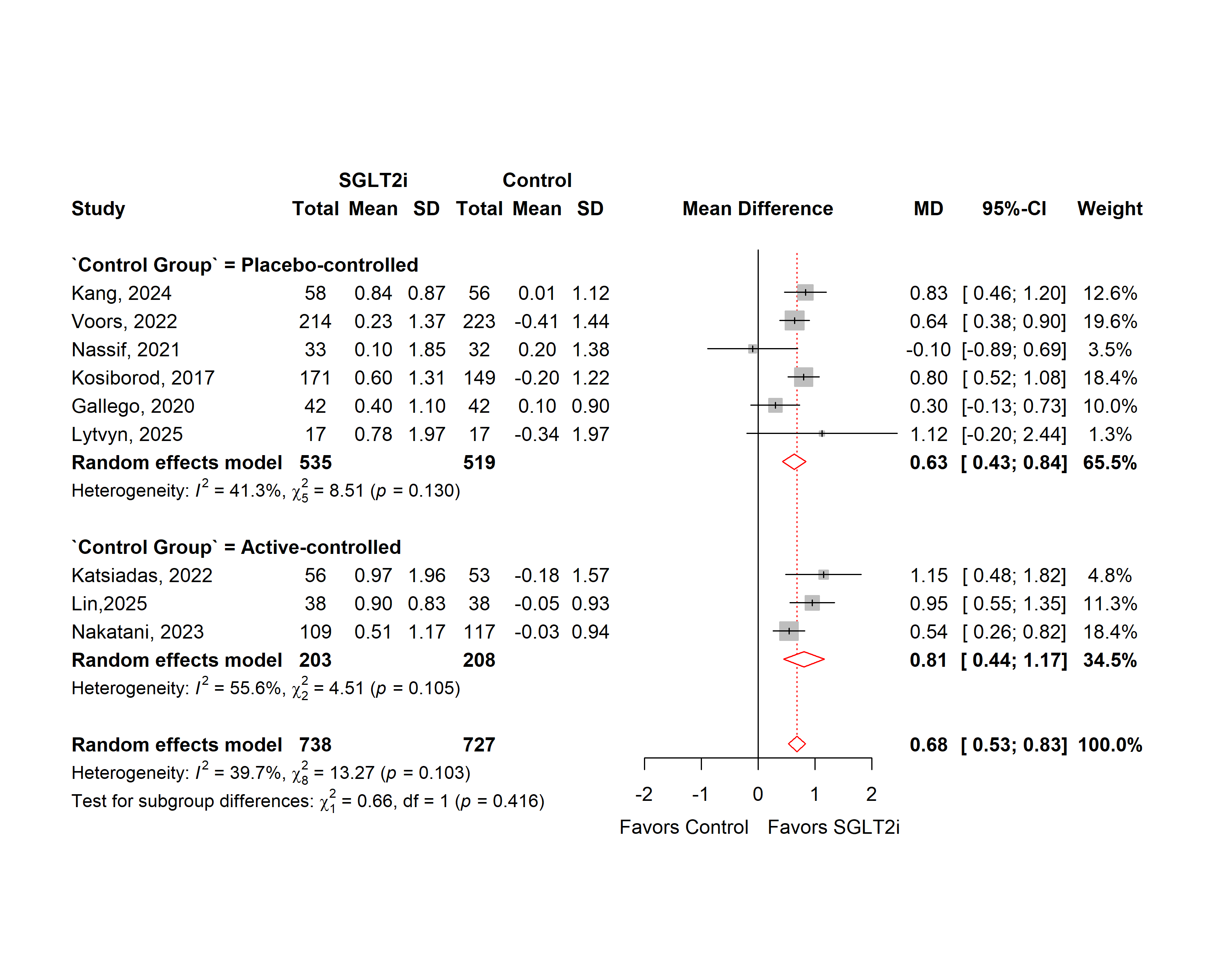


## Supplemental Figure S4: Subgroup analysis of SGLT2 inhibitors on hematocrit (Hct, %) changes by control group (placebo-controlled vs active-controlled): Meta-analysis of MD and 95% confidence intervals (CIs) across all drug groups using a random-effects model. CI: confidence interval; MD: mean difference.


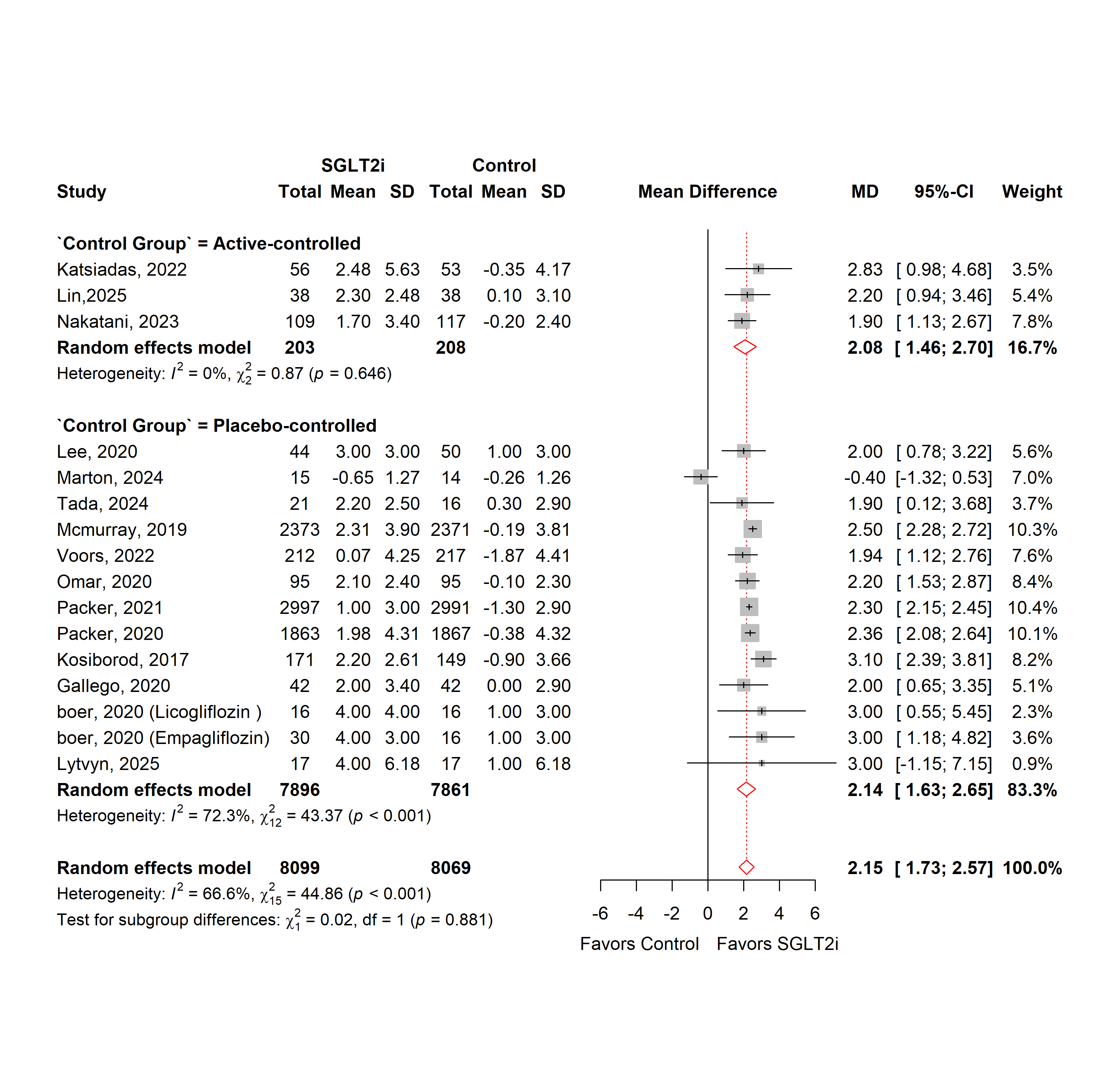


**Supplemental Figure S5:** **Meta-regression analysis examining the association between baseline hematocrit (Hct), Age, Body Mass Index (BMI), Creatinine (Cr), Diabetes Mellitus (DM), Hypertension (HTN), estimated Glomerular Filtration Rate (eGFR), Follow-up duration, Heart Rate (HR), Male sex, Ejection Fraction (EF), Angiotensin-converting enzyme (ACE) inhibitors or Angiotensin Receptor Blocker (ARB) use, Angiotensin Receptor Neprilysin Inhibitor (ARNI) use, Diuretics use and Hct response to SGLT2 inhibitors therapy**


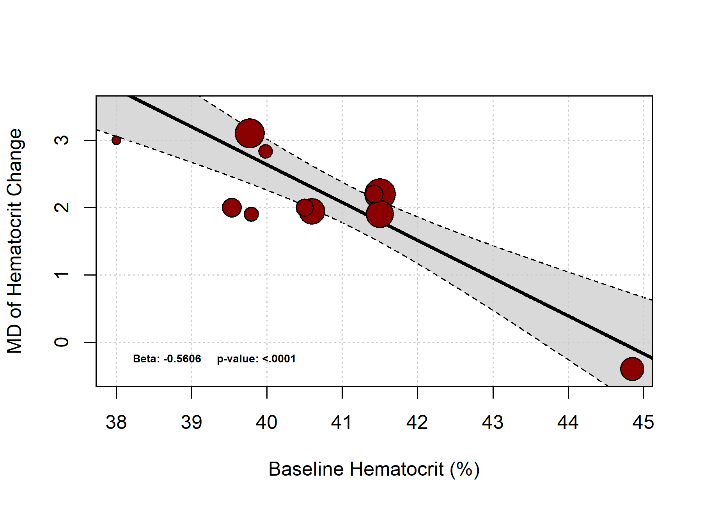

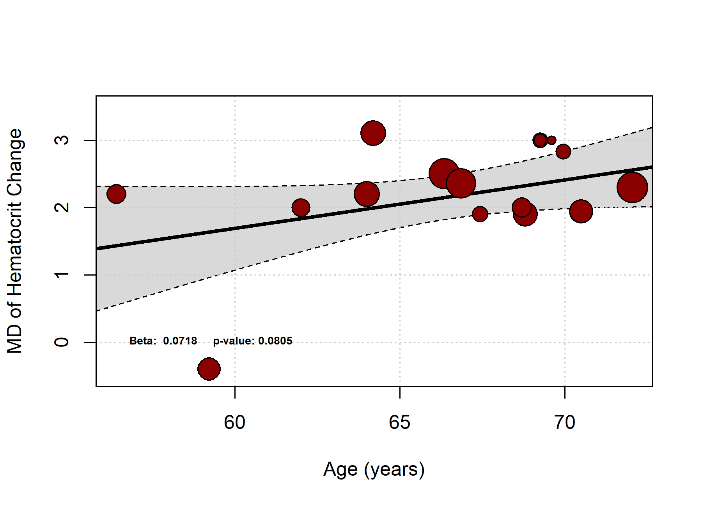

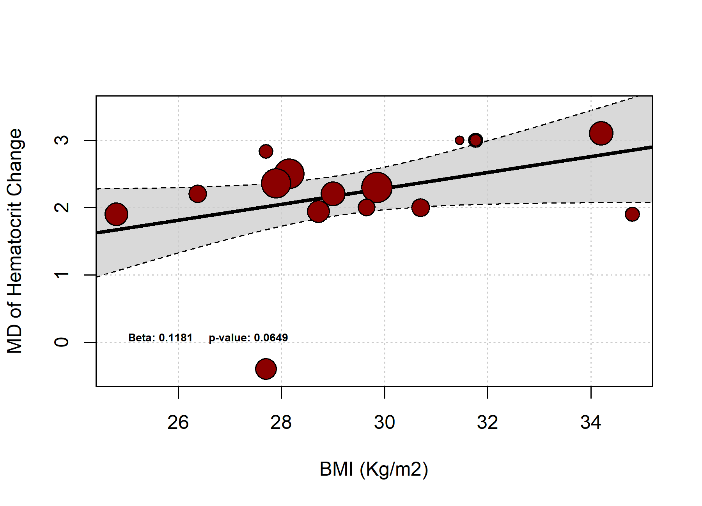

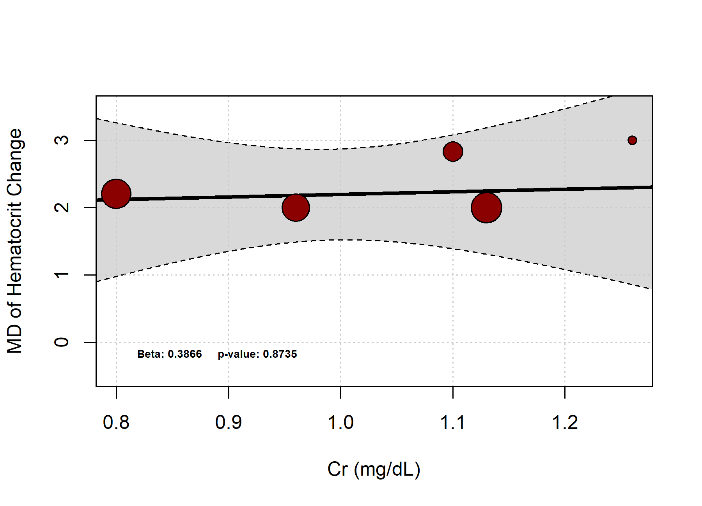


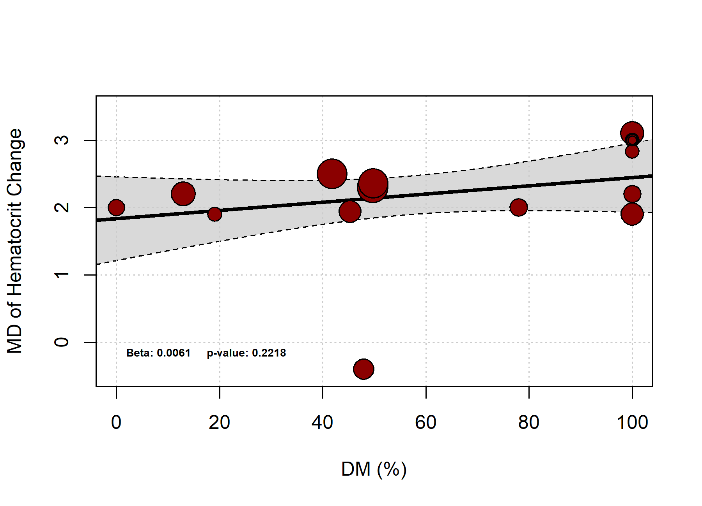

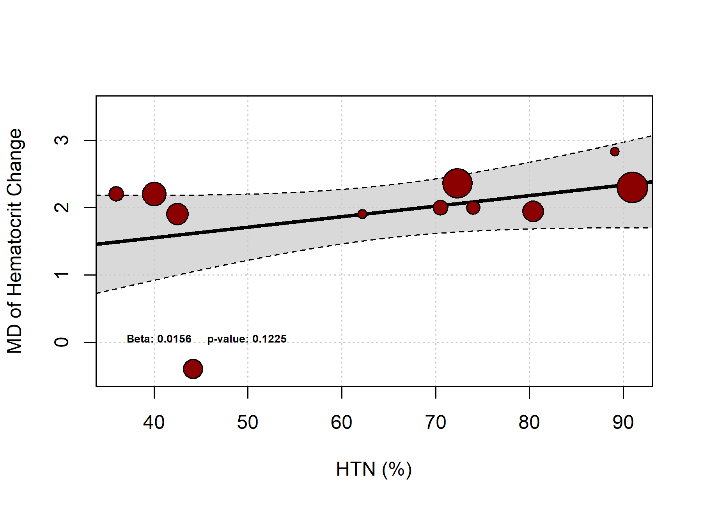


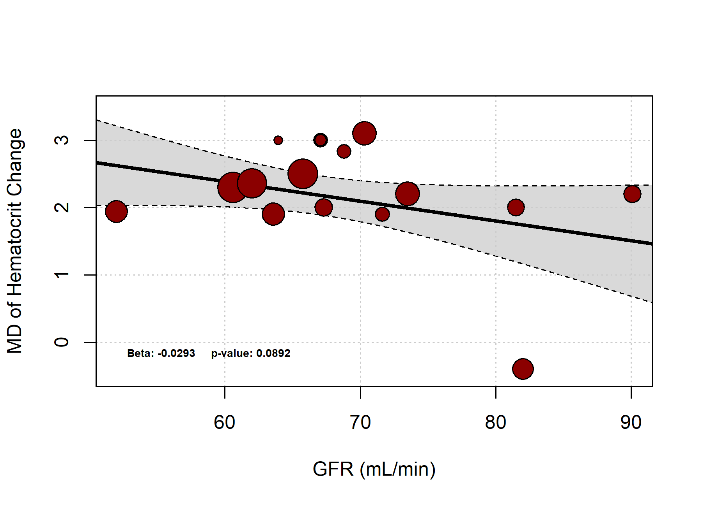

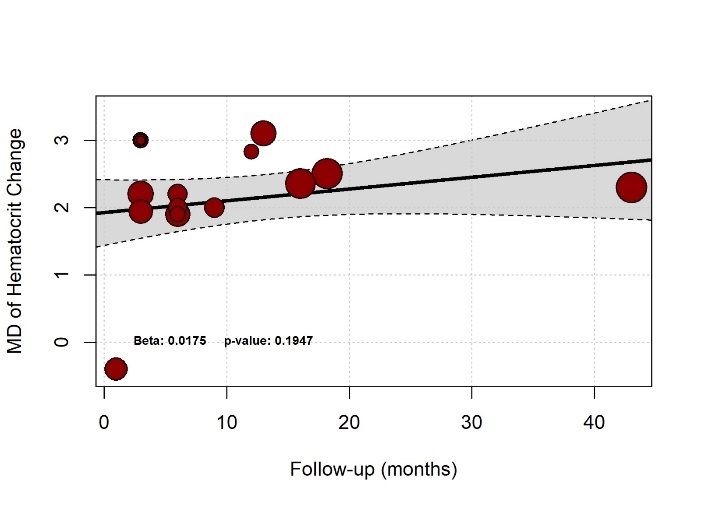


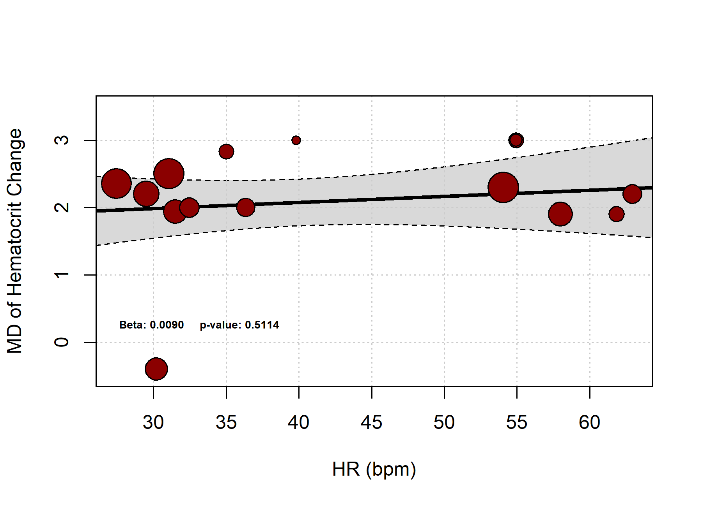

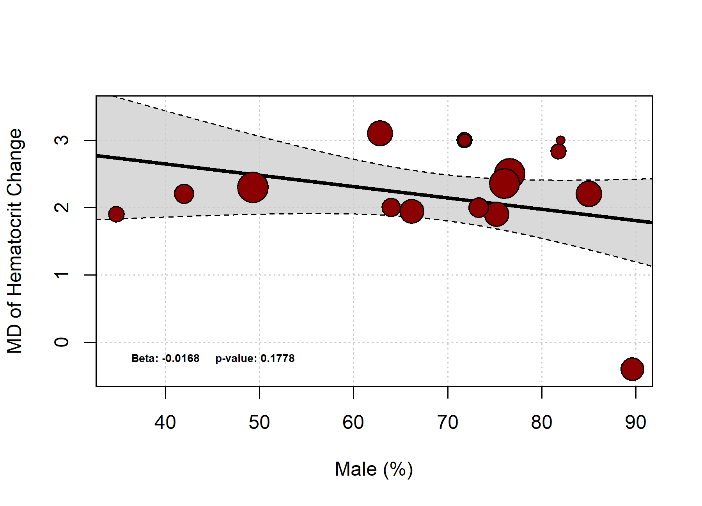


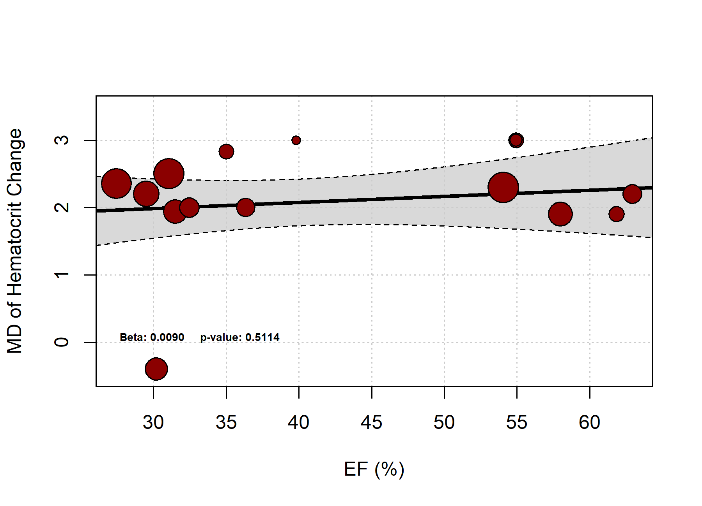

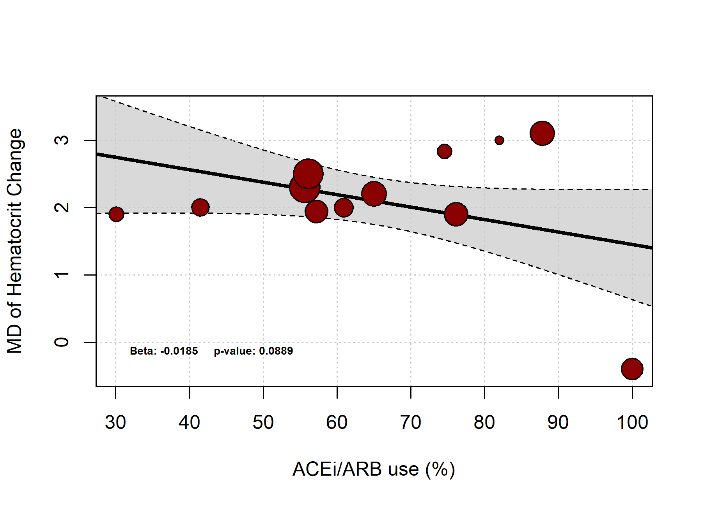


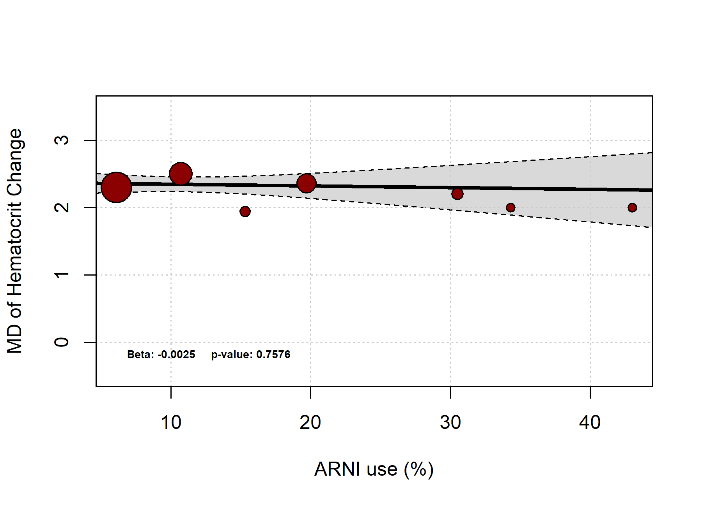

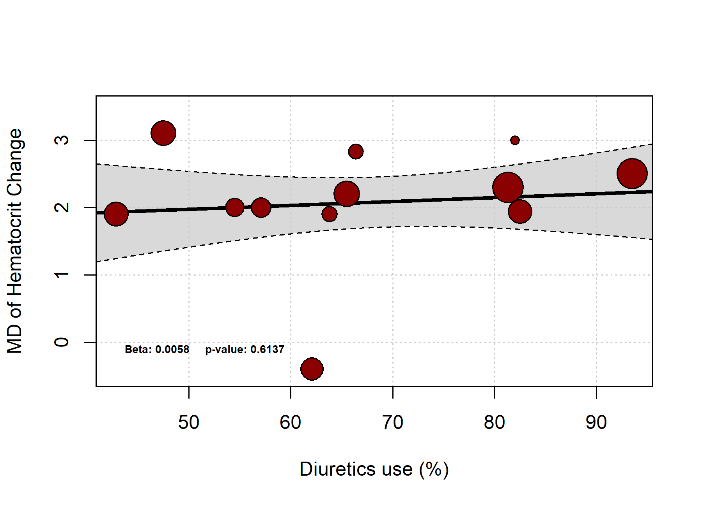


**Supplemental Figure S6:** **Meta-regression analysis examining the association between baseline hemoglobin (Hb), Age, Body Mass Index (BMI), Creatinine (Cr), Diabetes Mellitus (DM), Hypertension (HTN), estimated Glomerular Filtration Rate (eGFR), Follow-up duration, Heart Rate (HR), Male sex, Ejection Fraction (EF), Angiotensin-converting enzyme (ACE) inhibitors or Angiotensin Receptor Blocker (ARB) use, Angiotensin Receptor Neprilysin Inhibitor (ARNI) use, Diuretics use and Hb response to SGLT2 inhibitors therapy**


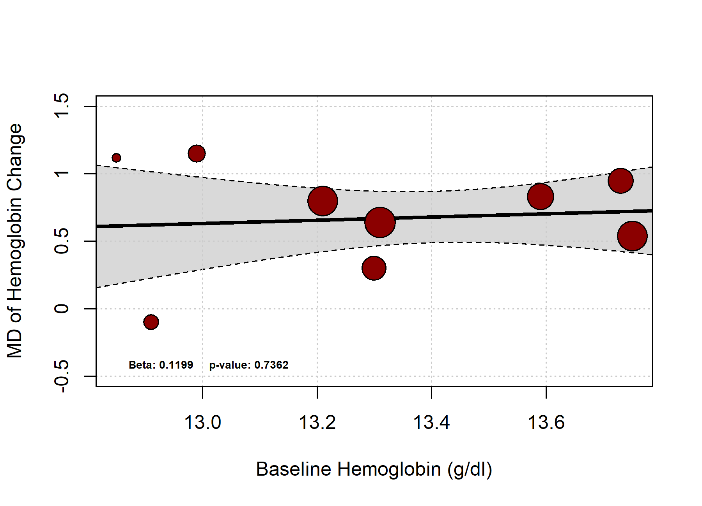

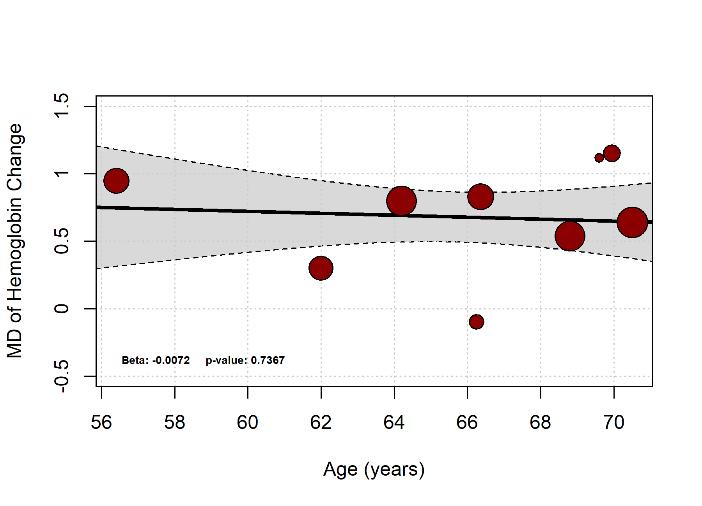


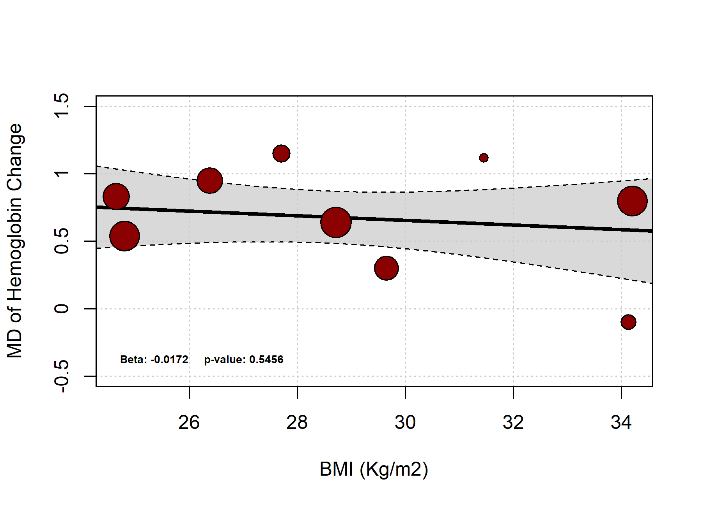

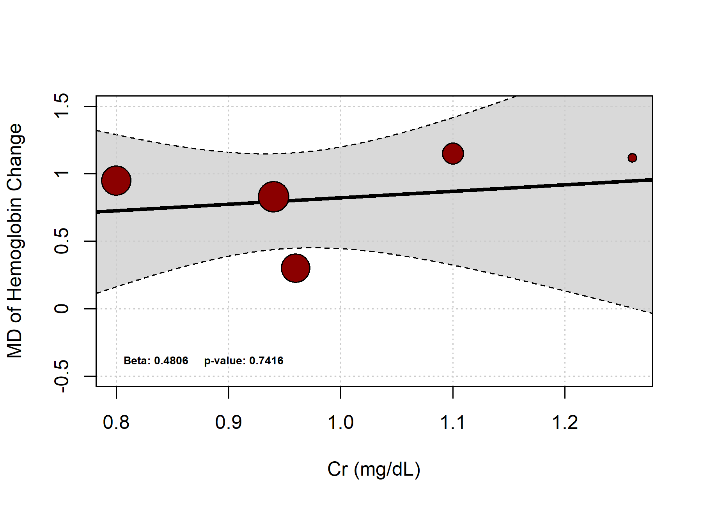


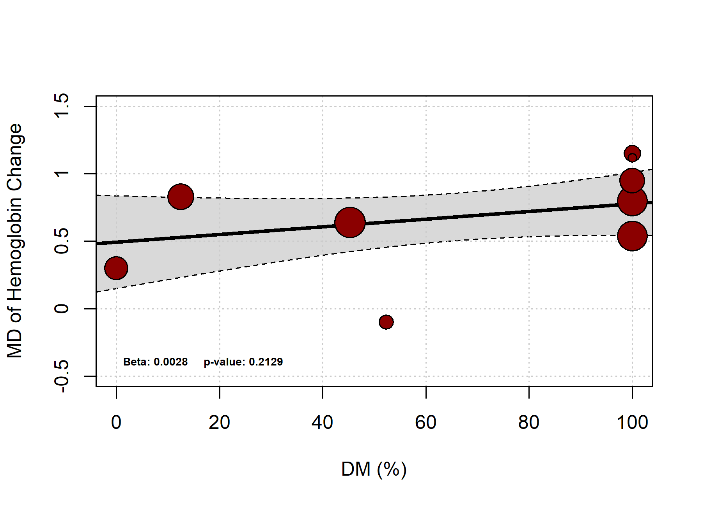

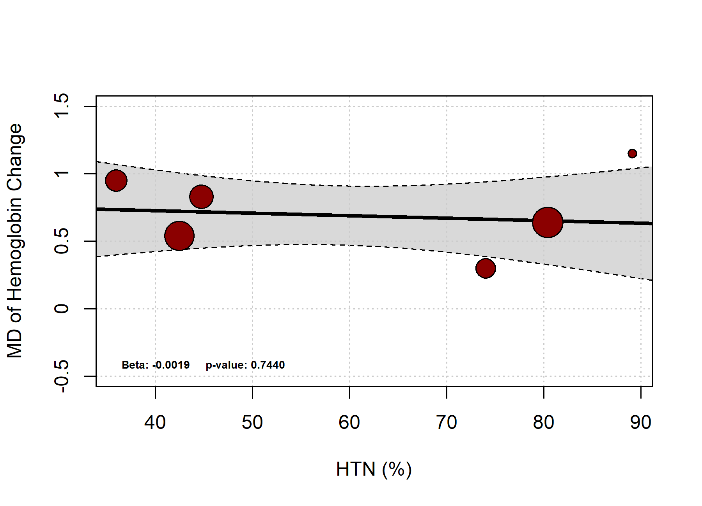


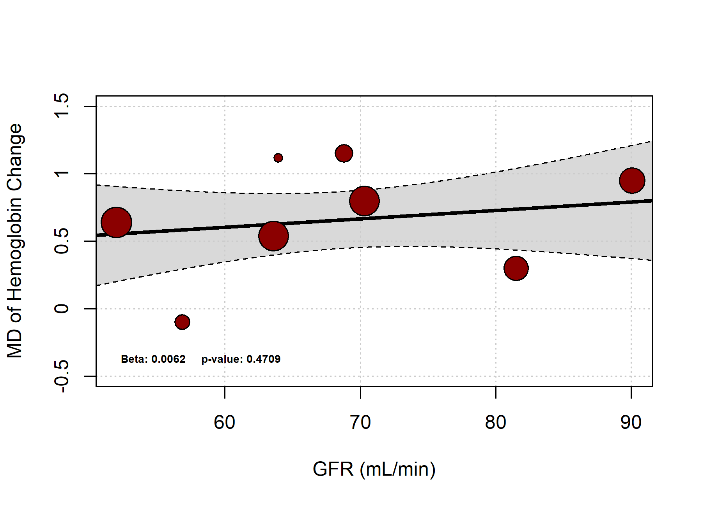

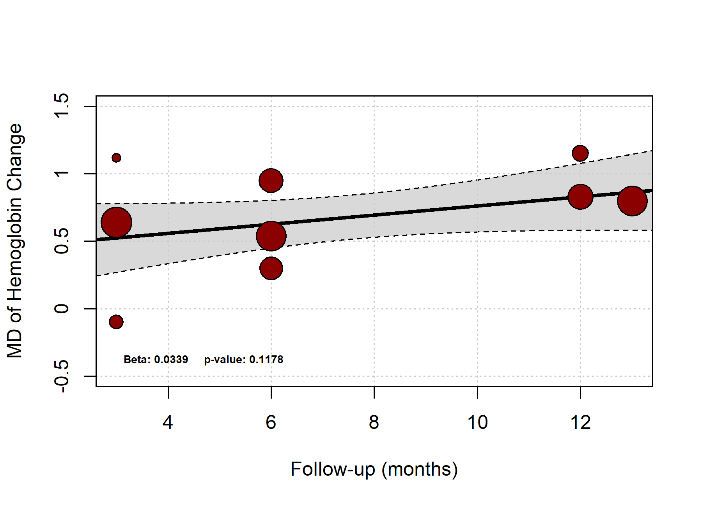


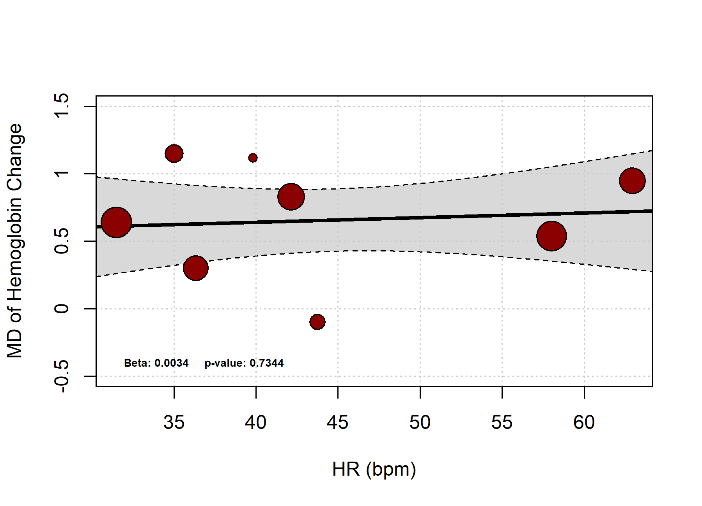

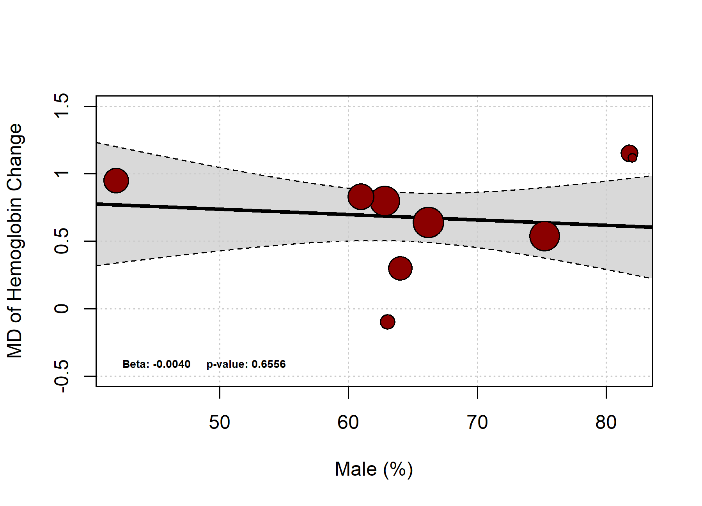


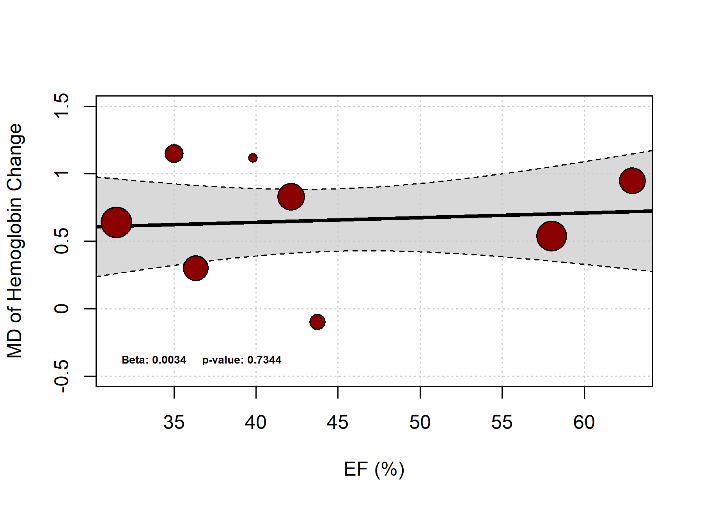

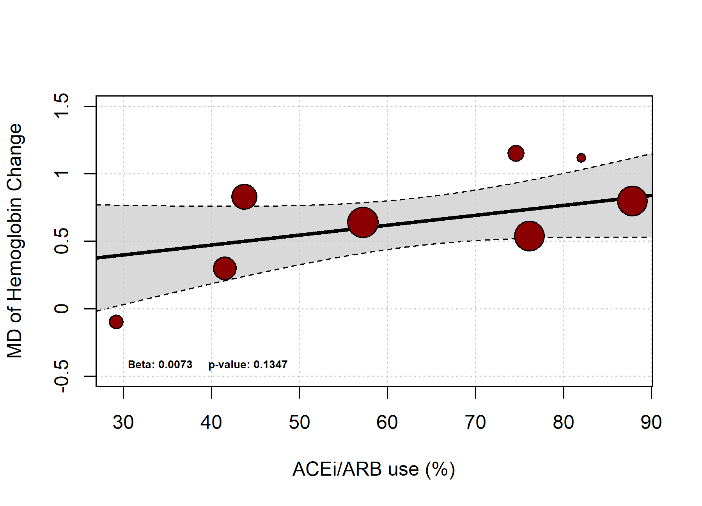


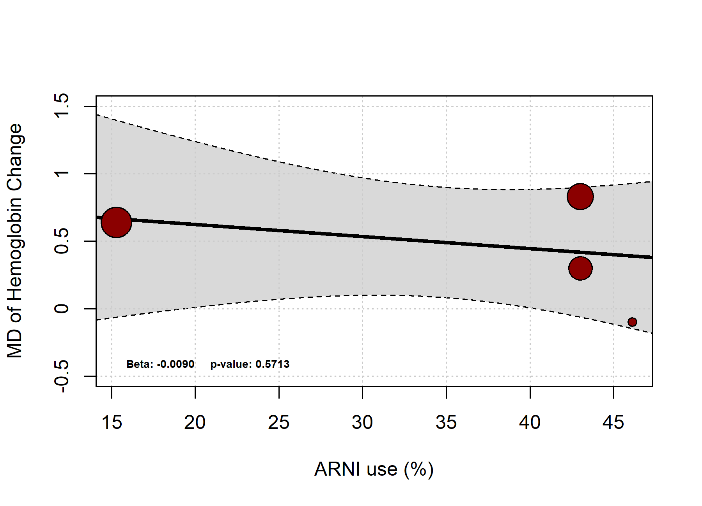

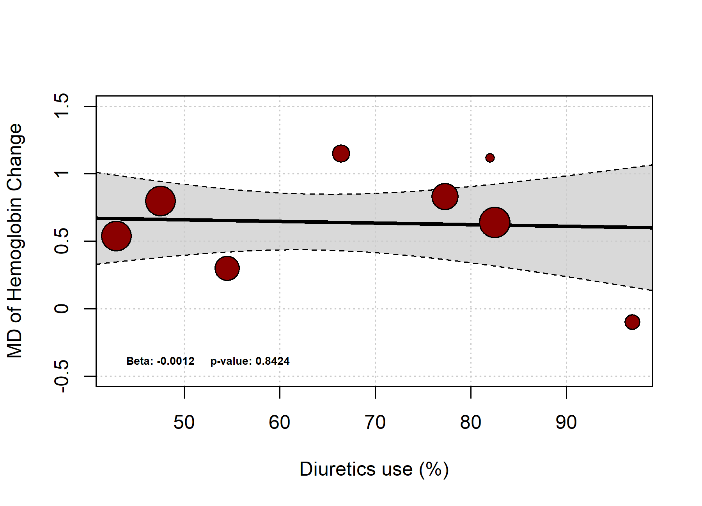


Supplemental Figure S7: Funnel plot of hemoglobin (Hb), a symmetrical funnel plot revealed no potential publication bias for the comparison of Hb levels between the intervention and control groups


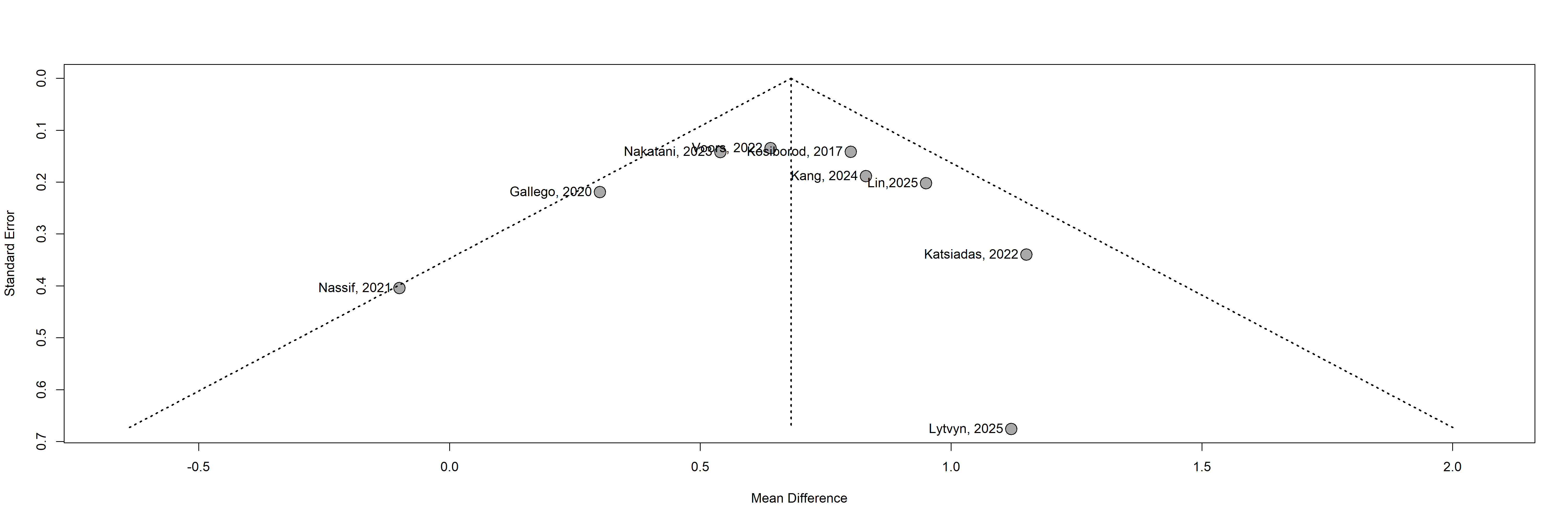


Supplemental Figure S8: Funnel plot of hematocrit (Hct), a symmetrical funnel plot revealed no potential publication bias for the comparison of Hct levels between the intervention and control groups


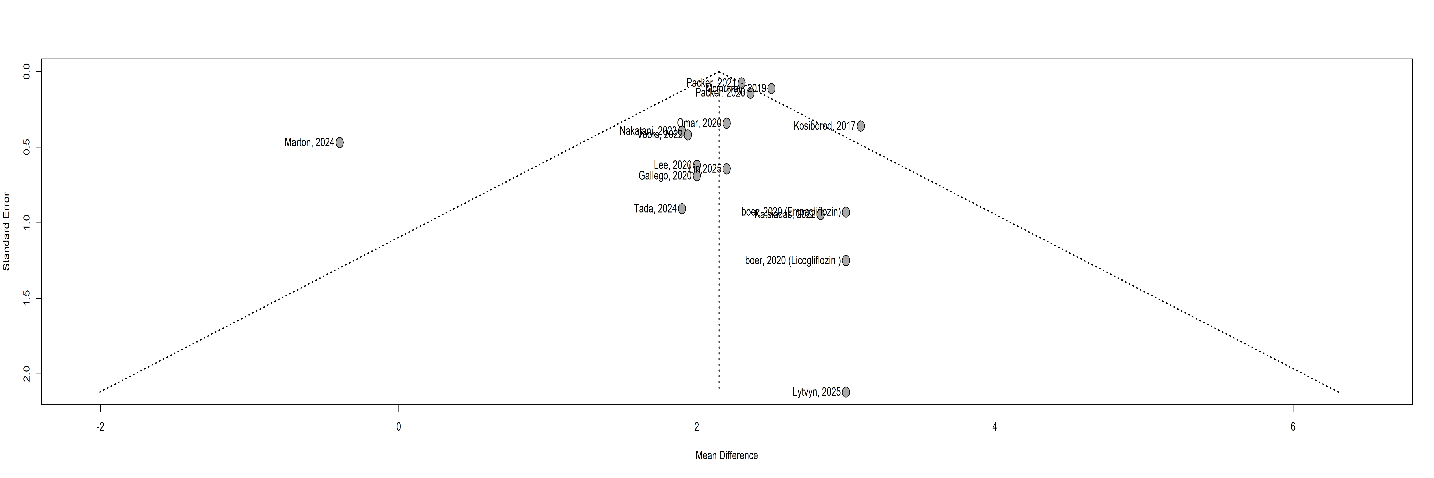

Supplement: xvag027_Supplementary_Data [file xvag027_supplementary_data.docx]
